# Utility of the Neonatal Calf Model for Testing Vaccines and Intervention Strategies for Use against Human RSV Infection

**DOI:** 10.3390/vaccines7010007

**Published:** 2019-01-08

**Authors:** Mariana Guerra-Maupome, Mitchell V. Palmer, Jodi L. McGill, Randy E. Sacco

**Affiliations:** 1 Department of Veterinary Microbiology and Preventative Medicine, Iowa State University, Ames, IA 50011, USA; marianag@iastate.edu (M.G.-M.); jlmcgill@iastate.edu (J.L.M.); 2 Infectious Bacterial Diseases of Livestock Research Unit, National Animal Disease Center, Agricultural Research Service, United States Department of Agriculture, Ames, IA 50010, USA; mitchell.palmer@ars.usda.gov; 3 Ruminant Diseases and Immunology Research Unit, National Animal Disease Center, Agricultural Research Service, United States Department of Agriculture, Ames, IA 50010, USA

**Keywords:** RSV, calf model, human, vaccines, therapeutics

## Abstract

Respiratory syncytial virus (RSV) is a significant cause of pediatric respiratory tract infections. It is estimated that two-thirds of infants are infected with RSV during the first year of life and it is one of the leading causes of death in this age group worldwide. Similarly, bovine RSV is a primary viral pathogen in cases of pneumonia in young calves and plays a significant role in bovine respiratory disease complex. Importantly, naturally occurring infection of calves with bovine RSV shares many features in common with human RSV infection. Herein, we update our current understanding of RSV infection in cattle, with particular focus on similarities between the calf and human infection, and the recent reports in which the neonatal calf has been employed for the development and testing of vaccines and therapeutics which may be applied to hRSV infection in humans.

## 1. Introduction

Respiratory syncytial virus (RSV) is the leading cause of lower respiratory tract infections in children in the first year of life [1]. Worldwide, RSV disease in children under the age of 5-years causes an estimated 33.8 million lower respiratory tract infections, and up to 200,000 deaths annually [2]. Human RSV (hRSV) is highly infectious; the majority of children have been infected with hRSV at least once by the age of three years [3]. Furthermore, in the elderly, hRSV has a disease burden similar to that of seasonal influenza A virus [4]. Despite the high burden, treatment is largely supportive, and there is no vaccine available to prevent or reduce disease.

Bovine RSV (bRSV) is the cause of enzootic pneumonia in young dairy calves and summer pneumonia in nursing beef calves. BRSV infection is associated with a high morbidity (60 to 80%) and mortality can reach up to 20% in some outbreaks. Moreover, bRSV is a major viral agent of the bovine respiratory disease complex (BRDC) in feedlot cattle. In the US, BRDC is responsible for estimated economic losses of $54.12 million annually, not including production losses due to morbidity and mortality [5]. Current bRSV vaccines available on the market are poorly effective, especially in young calves with maternally derived antibodies.

BRSV and hRSV are genetically and antigenically similar. The disease caused by bRSV in calves parallels that caused by hRSV infection in human infants in many aspects, including seasonal periodicity, immune response and disease pathology. The neonatal calf represents a tractable model of the infant immune system and is of scalable size to humans, thus representing a physiologic host model in which to study host antiviral immunity and viral pathogenesis, and to develop novel intervention strategies. Here, we update our current understanding of bRSV infection in cattle, with particular focus on similarities between the calf and human infection, and the recent reports in which the neonatal calf has been employed for the development and testing of vaccines and therapeutics which may be applied to hRSV infection in humans.

## 2. Age and Seasonal Affects

Most severe acute lower respiratory tract infections due to hRSV occur in infants and children less than 1 year of age, especially those born prematurely or with underlying cardiopulmonary conditions [4,6]. Reinfection can occur through life. By the age of two, the majority of children have experienced at least one infection [3]. In cattle, bRSV-associated disease is most pronounced in calves younger than 6 months of age. Based on surveys completed in the US, it has been estimated that more than 50% of calves have been exposed to bRSV by breeding age [7]. As with humans, re-infections in calves are common. Seasonal periodicity is seen with hRSV and bRSV, with most common occurrences from October through May [8].

## 3. RSV Viral Proteins

HRSV and bRSV (family *Paramyxoviridae*, subfamily *Pneumoviridae*, and genus *Pneumovirus*) are single-stranded, negative-sense viruses with a genome of approximately 15.2 kb. The RNA genome contains 10 viral genes encoding 11 proteins, with the M2 gene encoding two proteins. RSV proteins nucleocapsid (N), phosphoprotein (P), large polymerase (L), and associated proteins, transcriptional anti-termination factor M2-1 and RNA regulatory protein M2-2 are involved in nucleocapsid structure or synthesis of viral RNA. Transmembrane surface glycoproteins include cellular attachment (G), fusion (F), and small hydrophobic (SH). Matrix or membrane, M, is a non-glycosylated protein associated with the inner face of the envelope. Finally, there are 2 non-structural proteins NS1, NS2, which accumulate in infected cells. A recent review has nicely summarized interactions of these RSV proteins with the host immune response [9]. For purposes of this review, we will concentrate on those viral proteins primarily included in current experimental RSV vaccines in development or in early clinical trials.

Based on antigenic and genetic differences, hRSV has been classified into two subgroups, A and B [10,11]. While bRSV isolates can be classified into subgroups based on reactivity of mAb to the G protein [12], it has been suggested that these may represent variants of a single major antigenic group [12,13]. The F protein is a type I viral fusion protein synthesized as a metastable precursor (pre-F) that is proteolytically cleaved by furin into disulfide-linked F_2_ and F_1_ heterodimers, key for membrane fusion and viral entry [14]. There is more divergence in the type II integral membrane protein G than for the F protein among hRSV subgroups [15]. BRSV isolates possess antigenically heterogeneous G proteins, but the nucleotide sequences are less variable than for hRSV [7,12]. The predominant neutralization and protective epitopes are found in the G and F protein, with the most potent neutralizing antibodies targeting the pre-F form. The pre-F undergoes spontaneous structural rearrangements to the post-fusion (post-F) form. Post-F fails to present epitopes for many of the most highly potent neutralizing antibodies. The attachment G protein is a major target of the host antiviral antibody response for both hRSV and bRSV [16], indicating that specific regions of the protein may be under immune selection [17]. However, it has been suggested that differences in the variability of the central hydrophobic region of the G protein of these viruses could be indicative of differing immunological pressures between bRSV and hRSV [18]. HLA class I and II-restricted epitopes have been found in the N, M, NS2, M2-1, F and G proteins [19,20].

## 4. RSV Life Cycle

The RSV lifecycle has been extensively reviewed elsewhere [21,22], thus we provide only a brief overview of the lifecycle. There have been few comparisons of the lifecycle between bRSV and hRSV; however, it is likely that both viruses rely on similar mechanisms for their replication.

RSV enters the cell through fusion at the plasma membrane. The initiation step occurs when the G protein of the RSV tethers to the cell surface by binding candidate receptors such as TLR4, CX3CR1, or heparin sulfated proteoglycans [21]. Cell surface nucleolin may also be involved in the entry process by triggering fusion of the virus and host cell membranes by binding to RSV F. Host cell macropinocytosis of RSV is also a route of entry for RSV [21]. However, it remains to be elucidated which receptors are involved in this process. Viral gene expression and replication occurs in the cytoplasm. Internalization of the virion is dependent on actin rearrangement, phosphatidylinositol 3-kinase activity, and host cell early endosomal vesicles where proteolytic cleavage of the RSV-F protein triggers delivery of the capsid contents into the host cells by fusion of the virus and endosomal membranes [21]. Once internalized, RSV uncoats and capsid contents, nucleocapsid and the genome, are released. The non-essential M2-2 gene appears to govern the transition from transcription to production of genomic RNA, by downregulating transcription. The RNA polymerase complex (which includes the major subunit L) enters the genome at or near the 3′ end and genes are transcribed into separate, capped, polyA^+^ mRNAs by the start-stop-restart synthesis [22]. The P protein is an essential co-factor in RNA synthesis. A polar transcription gradient is created in which upstream genes are transcribed more frequently than downstream genes. Viral replication generates a complete positive-sense RNA complement of the genome called the antigenome, which acts as a template for genome synthesis. The genome and the antigenome are tightly encapsulated by the N protein which provides protected, flexible templates for RNA synthesis [22]. The inner envelope M protein regulates RSV morphogenesis by interacting with the transmembrane surface proteins F, G, SH, and with the nucleocapsid proteins N, P, and M2-1. The newly synthesized proteins then self-assemble, and budding occurs, with RSV viral envelope formed by association of M, and viral surface transmembrane proteins with the host cell plasma membrane lipid bilayer.

## 5. Experimental bRSV Infection in Calves

The clinical signs observed in experimental bRSV infection parallel that of hRSV and can vary from subclinical to severe, and sometimes fatal lower respiratory tract disease. In both human and cattle, clinical signs usually peak at 4 to 5 days post-infection and are manifested by nasal discharge, abnormal lung sounds, dyspnea, fever, hypoxia, and cough; with severe infection, interstitial pneumonia and bronchiolitis [23,24]. The severity of clinical disease and lung pathology in calves after experimental bRSV infection is influenced by the virulence of the viral isolate, dose of inoculum, route of infection (nasal, intratracheal, aerosol or a combination), age of calves, and presence of maternal derived antibodies. Exacerbated respiratory disease, similar to that seen in infants vaccinated with formalin inactivated (FI)-hRSV, has also been reproduced experimentally in calves [25].

Studies in human infants are hampered by both ethical and technical hurdles. As a natural pathogen of cattle, bRSV mimics hRSV pathogenesis more closely than experimental infection of semi-permissive laboratory animals. The large size of calves, even in neonates, allows for frequent collection and large volumes of blood, mucosal secretions (i.e. nasal fluid), and bronchoalveolar lavages (BAL), to analyze kinetic changes in the peripheral and local immune responses to infection and vaccination (Table 1). Further, analysis of pulmonary function frequently used in humans, can be applied to calves during experimental bRSV infection [26,27]. Finally, calves receive maternal antibodies through the ingestion of colostrum, this feature allows selective deprivation of maternal antibodies; thus, eliminating confounding effects of maternal antibody on clearance of bRSV. Therefore, bRSV infection in the calf represents a valuable model to study the pathogenesis and immunity to hRSV infection in children, as well as for preclinical testing of vaccines and therapeutics.

## 6. Histopathology of Experimental bRSV Infection and Similarities to Lesions in Human Infants

We have used bRSV strain 375 for inoculation of neonates and young animals in our studies [32,47,48]. Virus stock re-isolated from the lung of an infected animal and passaged less than 4 times on bovine turbinate cells was used to prepare a standardized inoculum. Our bRSV aerosol challenge model in calves [32] uses a protocol similar to that described by Woolums et al. [49]. Briefly, the nebulized challenge inoculum is delivered via a mask covering the nostrils and mouth. The aerosol system consists of a compressed air tank, a jet nebulizer, and a mask (Trudell Medical International, London, ON, Canada) which has been modified to fit calves. Jet nebulization of the challenge inoculum occurs in a holding reservoir using compressed air delivered at 25 lb/in^2^. The nebulized inoculum is inhaled through a one-way valve into the mask and directly into the nostrils. Each calf receives a challenge inoculum containing approximately 5 × 10^4^ TCID_50_ of bRSV strain 375 delivered during a nebulization period of 10–15 min.

**Figure 1:**
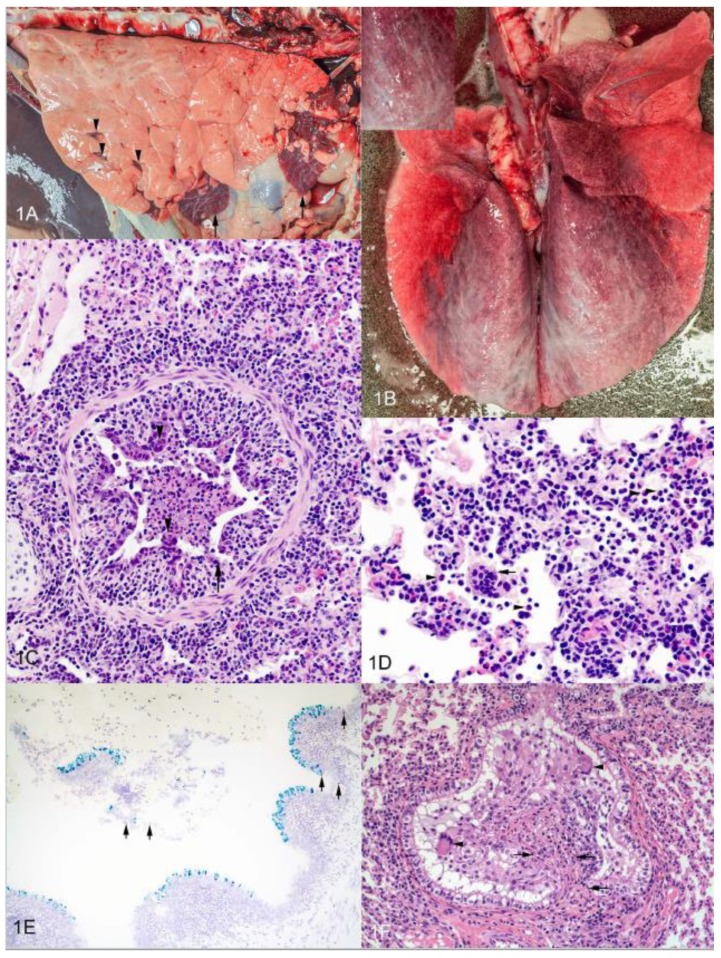
Gross and microscopic pathology of experimental BRSV infection. (**A**) Lung from calf experimentally infected with bRSV and examined 7 days later. Ventral areas of right cranial and right middle lung lobes are atelectic and dark red-purple (arrows). Similar small lesions are visible in the right caudal lobe (arrowheads). The remainder of the lung failed to collapse. (**B**) Lung from calf experimentally infected with bRSV and examined 7 days later. There is an overall red discoloration to the lungs. Dorsal regions of all lobes are characterized by interlobular and subpleural edema (inset). (**C**) Photomicrograph of lung from calf experimentally infected with bRSV and examined 7 days later. There is degeneration and necrosis of bronchial epithelium with sloughed cells and debris in bronchial lumen. Some areas lack epithelium (arrows). Note epithelial syncytia (arrowheads). (**D**) Photomicrograph of lung from calf experimentally infected with bRSV and examined 7 days later. Interstitial capillaries are congested and alveolar interstitium contains numerous mononuclear cells. Alveolar lumina contain macrophages, neutrophils (arrowheads) and syncytia (arrow). (**E**) Photomicrograph of lung from calf experimentally infected with bRSV and examined 7 days later. In situ hybridization using probes for bRSV F protein (green) and IL-8 (red). (**F**) Photomicrograph of lung from calf experimentally infected with bRSV and examined 14 days later. Bronchiolitis obliterans; polypoid proliferative epithelium, including syncytia (arrowheads), supported by a fibrous stalk (arrows) fills bronchiolar lumen.

In the lungs of experimentally infected animals, gross lesions of bRSV infection are often cranioventral in distribution and characterized by multifocal to focally extensive atelectatic, deep red-purple lesions that are rubbery upon palpation. However, similar smaller red-purple lesions surrounded by lobules of pink, hyperinflated lung may be present in the caudal, middle and accessory lobes as well (Figure 1A) [32]. Caudodorsally, lobes may fail to collapse and be distended by interlobular or subpleural edema and/or emphysema. In some variations of gross appearance there may be an overall red discoloration of the lung (Figure 1B). The nasal meatus, trachea, bronchi and bronchioles can contain foamy to mucopurulent discharge. Pulmonary lymph nodes (i.e., mediastinal and tracheobronchial) may be enlarged, edematous or even hemorrhagic.

Microscopic lesions are characterized as bronchointerstitial pneumonia, necrotizing bronchiolitis, syncytia formation, type II pneumocyte hyperplasia and exudative or proliferative alveolitis. Necrotizing bronchiolitis, the most consistently observed finding, characterized by a degenerative or necrotic bronchiolar epithelium, leads to ulcerated foci covered by necrotic cellular debris with nearby degenerate cells displaying rounded cell borders. Due to ulceration, epithelial cells may be attenuated as they extend over exposed basement membrane (Figure 1C); resulting in bronchioles lined by flattened cells having a squamous morphology. Bronchiolar epithelial syncytial cells projecting from the bronchiolar wall or free in the lumen may be observed (Figure 1D). Less commonly, syncytial cells may also be found in alveoli. Uncommonly, eosinophilic intracytoplasmic viral inclusions may be seen in syncytial cells, mononuclear leukocytes or epithelial cells. Neutrophilic exudates, fibrin and cell debris may be present within bronchi, bronchioles and alveoli (Figure 1D). Nearby alveoli may be modestly dilated due to hyperinflation. Variable thickening of the alveolar interstitium is seen; the result of alveolar capillary congestion, edema, lymphangiectasia, infiltrates of lymphocytes and macrophages, and type II pneumocyte hyperplasia. The majority of the tissue damage is thought to be the result of host response rather than primary viral infection since lesion severity frequently does not correlate with viral load or viral distribution. Immunohistochemistry [50] or in situ hybridization may reveal viral antigen or RNA in bronchiolar epithelial cells (Figure 1E). Tracheobronchial lymph nodes of affected cattle are often found with enlarged cortical areas containing prominent follicles and expanded parafollicular areas and medullary cords due to lymphocytic hyperplasia. Medullary sinuses frequently contain macrophages, variable numbers of lymphocytes and plasma cells, and occasionally neutrophils and eosinophils.

As viral infection progresses, an attempt to repair necrotic airway epithelium may result in epithelial hyperplasia and bronchiolitis obliterans, also known as bronchiolitis fibrosa obliterans, obliterative bronchiolitis or organizing bronchiolitis [51] and has been characterized as “wound healing gone awry” [51]. Persistent fibrinous and exudative inflammation is followed by fibroblast infiltration and neovascularization in an attempt at healing. In as little as 10 days after infection, there may be fibrous polyps, covered by respiratory epithelium that extend into the airway lumen often resulting in permanent decreases in airflow and alveolar ventilation. In our young calf model, we have seen evidence of bronchiolitis obliterans 14 days after experimental infection (Figure 1F). Bronchiolitis obliterans is not specific for bRSV and can be induced by various agents (e.g., viral, bacterial, parasitic, toxic). Ultrastructurally, changes in ciliated and non-ciliated epithelial cells, as well as both type I and type II pneumocytes can range from acute cell swelling with expanded intercellular areas and loss of intercellular junctions to necrosis [52].

Where histopathological evaluation is available from fatal hRSV cases, it was observed that airway lumina contain leukocytic and sloughed epithelial cell debris, with fibrin and a minor amount of mucin [53,54,55]. Further obstruction of the airways was due to submucosal edema and peribronchiolar infiltrates, consisting predominately of mononuclear cells with few neutrophils. With interstitial pneumonia, there was evidence of significant alveolar involvement including edema and cellular infiltration, as well as epithelial cell attenuation or loss.

## 7. Innate Immune Response to bRSV and hRSV

BRSV and hRSV replication starts in the upper respiratory tract epithelial cells followed by replication in bronchial and alveolar epithelium [56]. Innate immune receptors on epithelial and lung sentinel cells, including Toll-like receptors (TLRs), retinoic acid-inducible gene I-like receptors (RLR), and nucleotide-binding oligomerization domain (NOD)-like receptors recognize virus-specific structural components, leading to the production of proinflammatory cytokines and chemokines that serve to orchestrate anti-viral immunity (reviewed [57]). Increased expression of a wide range of proinflammatory cytokines and chemokines including RANTES (CCL5), MIP-1α (CCL3), MCP-1 (CCL2), IL-8 (CXCL8), IL-6, IL-12, IL-1β, IL-18, IL-10, IFN-γ and TNFα have been detected from bRSV-infected calves (Table 1) [26,32,58,59]. The mechanisms involved in the induction and regulation of these immune modulators are not clearly defined; however, increased secretion contributes to the airway damage caused by both bRSV and hRSV infection. For instance, in humans the development of maximum clinical severity and viral load (peaking on days 4–7), coincides with an increased RANTES/IL-8 ratio and maximal neutrophil response [34,39,60]. In accordance, we have previously shown that clinical signs of bRSV infected cattle develop in parallel to a pulmonary neutrophil response, and upregulation of IL-8 in infected lungs, at 5–7 days post-infection (Figure 1E) [32,38]. IL-8 is associated with the pathogenesis of bronchiolitis [61], which is typically seen in both bRSV and hRSV infection. Although, production of IL-8 in the lung has not been studied extensively during bRSV infection, the neonatal calf model can prove useful as an in vivo platform for future exploration into pathways that specifically regulate IL-8 responses to RSV infection.

A number of TLRs have been linked to hRSV infection, including TLR2, TLR3, TLR4, and TLR7. Ligation of TLR4/CD14 complex by the hRSV F protein, results in increased TLR4 expression, activation of NF-kB, and the production of proinflammatory cytokines TNFα, IL-6 and IL-12 [62]. Although it has not been extensively studied, it is possible that TLR4 is also involved in the recognition of bRSV, as in vitro infection of bovine TLR4 transfected cells induces TLR4-dependent NF-κB response [63]. Recognition of dsRNA by TLR3 and RLR, and ssRNA by NOD2, TLR7, TLR8 activates NF-κB and interferon regulatory factors (IRFs) [64,65,66]. IRF3 and 7 control the expression of type I IFN (IFNα and IFNβ). These cytokines bind to the type I IFN receptor (IFNAR) to activate interferon stimulated genes (ISG) via JAK/STAT pathway that help restrict viral replication.

As a counter-strategy to the IFN type I response, bRSV and hRSV have a variety of immune modulatory and evasion strategies to promote virus infection and replication. Nonstructural proteins, NS1 and NS2, antagonize type I IFN by inhibiting activation of IRF3 in a species-specific manner in infected epithelial cells and antigen presenting cells (APCs) [67,68,69]. In the case of bRSV NS proteins interfere in type I signaling via blocking phosphorylation and activation of IRF3 [68]. By comparison, human NS proteins inhibit STAT2 signaling to suppress IFN response [70]. Further studies have demonstrated that the hRSV NS genes may also play a role in activation of NFκB [71]; however, the role of bRSV NS proteins in NFκB activation has not been explored. Although NS proteins are not essential for RSV replication, single or double deletions of the NS genes (ΔNS1, ΔNS2 and ΔNS1NS2] show reduced ability to replicate in vitro and in vivo [72,73]. These observations suggest that inhibition of type I IFN signaling results in modulation of immune response genes, which counteracts the defense responses against both hRSV and bRSV and allows the establishment of a productive infection.

Antigen presenting cells (APCs), are major producers of cytokines that modulate downstream immune responses. The SH protein is transmembrane pore-forming protein that has the ability to negatively regulate the APC function by modulating secretion of proinflammatory cytokines. In vitro infection with recombinant bRSV or hRSV, in which the SH gene has been deleted, results in increased apoptosis, and increased secretion of IL-1β and TNFα by bovine and human epithelial cells and by bovine monocytes, compared to wild-type virus [74,75]. Further, in vitro studies have demonstrated that in contrast to SH deficient bRSV, SH expression in wild-type bRSV results in downregulation of proinflammatory cytokines, IL-1β and TNFα, in APCs through the selective inhibition of NFκB p65 phosphorylation by blocking the degradation of IkBa [76]. Similar to observations using rhRSVΔSH vaccine in chimpanzees, mucosal vaccination with rbRSVΔSH in calves, results in site-specific attenuation in the lungs, and long-lasting protective immunity against virulent challenge [74,75]. Thus, the SH protein counteracts the antiviral effects of the immune response by inhibiting early innate immune response.

Proteomic analysis of samples collected from the lower respiratory tract of bRSV infected calves found that the number of neutrophils in the lower respiratory tract and/or the relative expression of neutrophil-related proteins strongly correlates with gross pathology, clinical signs and viral shedding [77]. The histopathological hallmark of RSV infection is characterized by airway obstruction from neutrophils, sloughed bronchial epithelial cells and mucus plugs containing DNA networks in the form of neutrophil extracellular traps (NETs). RSV F protein induces NETs through activation of TLR4 or Fcγ receptors [78]. NETs serve to trap and kill microbes, but additionally adversely increase the viscosity of mucus. The presence of proteins associated with NETs, during bRSV infection, agrees with previous findings seen in lung pathology during severe hRSV-infected infants [79]. These results suggest that NETs induce lung injury rather than suppress viral replication. Hence, strategies which aim to suppress neutrophil-related proteins could potentially prevent clinical signs, and excessive pulmonary inflammation in both bRSV and hRSV infections.

## 8. Adaptive Immune Response

A dysregulated immune environment due to an imbalance in the Th1 and Th2 responses is thought to underlie the disease pathology described previously in this review (Section 5). Despite eliciting the production of neutralizing antibodies and virus-specific T cells, immunity to both hRSV and bRSV appears to wane and individuals remain susceptible to reinfection throughout their lifetime. Further, pulmonary pathology following severe hRSV may be a factor in airway reactivity and wheezing, causing prolonged episodes of asthma in infants [80].

### 8.1. Humoral Immunity to hRSV and bRSV Infection

Neutralizing antibodies can protect from severe hRSV disease as demonstrated by the efficacy of Palivizumab, a monoclonal antibody against the F protein, administered to premature and other high-risk infants [81]. Maternally derived antibodies do not protect against human or bovine RSV infection but reduce the severity of respiratory disease [40,82,83]. It has been demonstrated that maternal antibodies might suppress the development of both serum and mucosal antibodies and T cell responses [40,84].

**Figure 2:**
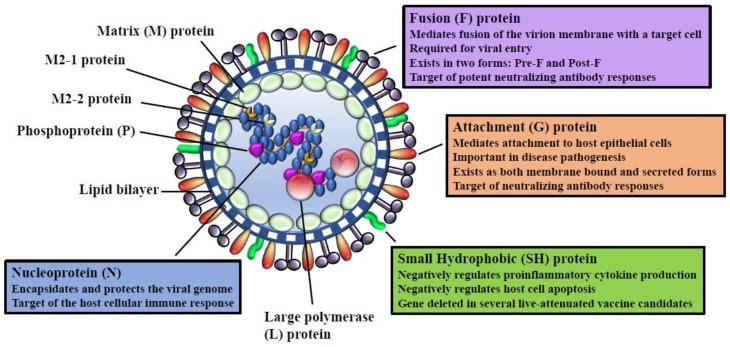
Schematic diagram of the RSV virion. Shown is the structure and organization of the RSV virion, emphasizing viral proteins targeted by the adaptive immune system.

The bRSV-specific B cell response is broad. Only the antibodies directed against the F and G appear to be important in protection [85]. Figure 2 highlights the major RSV proteins that are targeted by the adaptive immune system. The bRSV and hRSV G protein share only 30% amino acid identity, although both viruses share similar features. The G protein is a major target of the neutralizing antibody response, however, is antigenically variable in both viruses, thus, few antibodies cross-react between virus strains [20]. The G protein contains a central core domain that is highly conserved between hRSV strains and between bRSV. The conserved central core is an important target for neutralizing antibodies. G-specific neutralizing antibodies reduce RSV pathogenesis and viral burden in mice models, and elevated levels are associated with reduced severity scores in human patients [86]. Reports in calves have also shown that immunization with bRSV G elicit G protein specific antibody and T cell response and are protective against bRSV infection, as demonstrated by reduction of viral shedding and associated lung pathology [85,87,88,89].

On the other hand, the F protein is highly conserved, and is also a major target for neutralizing antibodies [20]. The F protein exists in two forms: a metastable pre-F form and a stable post-F trimer. Post-F contains two major neutralizing epitopes, antigenic sites II and IV [14]. In small animal models, post-F vaccines elicit high neutralizing antibodies against site II [90,91]. The pre-F form contains site II and Ø. Neutralizing antibodies specific to site Ø are potent and are a major target of the hRSV-specific neutralizing antibody response in adult humans [92]. Similar to observations in small animal models, vaccination of calves with the pre-F form induced >100 higher neutralization titers compared to the post-F. After bRSV challenge, pre-F immunization conferred superior protection against clinical signs, pulmonary inflammation and reduced bRSV replication in both the upper and lower respiratory tract [93]. Moreover, in bRSV seropositive animals a single administration of non-adjuvanted pre-F form markedly boosted pre-existing bRSV neutralizing responses and pre-F specific neutralizing antibodies, while post-F did not [94]. Therefore, pre-F represents a promising candidate for further increasing neutralizing antibodies in pre-exposed populations.

Reports describing the humoral response to the N protein are scarce. Immunization with vaccinia virus expressing the bRSV N protein induced non-neutralizing antibodies and primed N-specific T cell responses that resulted in lung protection [85]. Similarly, DNA vaccination with the bRSV N protein co-administered with plasmid DNA encoding the F protein resulted in low antibody levels, but primed bRSV-specific lymphoproliferative responses and IFNγ production in vitro and in vivo. This DNA vaccine reduced viral burden in the upper and lower respiratory tract of calves, clinical scores, and pulmonary lesions after virulent bRSV challenge. However, the role of F and N viral immunogens in protection was not investigated [95].

Neutralizing antibodies have an important role in protection from RSV infection, although serum and mucosal neutralizing antibodies seem to provide different levels of protection. Mucosal immunity is important in the upper airway, as anti-hRSV IgA levels in the nasal mucosa correlate with protection from infection [96]. BRSV-specific IgM and IgA can be detected in the nasal secretions and serum of bRSV infected calves as early as 8 days post-infection [40]. The ability to mount a rapid secondary mucosal IgA response plays more important roles in protection in the upper airways regardless of levels of serum neutralizing antibody, and correlates with recovery [40]. However, IgA responses wane and the neutralizing IgG response may be more important for long-term protection [97]. In children, protective antibody responses develop slowly [98]. Similarly, in calves, neutralizing IgG2 (associated with Th1 response) is undetectable until 1–3 months after infection. By comparison, IgG1 (associated with a Th2 response) is detectable 13 days post-infection [40]. This suggests that similar to hRSV disease in humans, bRSV infection may also be biased away from Th1, towards a dysregulated Th2 response.

### 8.2. Cellular Immunity to hRSV and bRSV Infection

Although antibody responses are vital for protection against both bRSV and hRSV, once infection is established, T-cell-mediated cellular immune responses have a greater role in virus clearance. Bovine and human CD8 T cells, recognize MHC class I epitopes in N, F, M, and M2 proteins, which are highly conserved between both viruses. Interestingly, G-specific CD8 T cells are found in cattle but have not been demonstrated in humans [99,100]. Cytotoxic CD8 T cells play an important role in viral clearance by secretion of cytokines IFNγ, TNFα, and IL-2 and the lysis of infected host cells [99,101]. Following bRSV and hRSV infection, virus-specific CD8 T cells expand in both frequency and total number in the lungs and airways 7–10 days after infection, which coincides with time of recovery and viral clearance [30,33,101,102,103]. In calves, depletion of CD8 T cells results in more prolonged virus shedding, and more severe pathologic lesions [30]. In humans, the role of CD8 T cells remains less clear; however, a paucity of CD8 T cells has been noted in lung tissue of infants that died of severe hRSV disease [28]. Further, children with T cell defects suffer more severe disease and prolonged viral shedding [104].

Based on the expression of activation markers and lymphoid homing receptors, memory CD8 T cells have been broadly separated into four major subsets: (1) naive (CD45RO-CCR7+), (2) central memory (T_CM_; CD45RO+CCR7+), (3) effector memory (T_EM_; CD45RO+CCR7−), and (4) late effector memory (T_EMRA_; CD45RA+CCR7−) [63]. T_CM_ home primarily to secondary lymphoid organs, while T_EM_ migrate to peripheral tissues and rapidly exert effector functions. Previous studies in infants with severe hRSV disease found that hRSV-specific CD8 T cells in blood and BAL are CD45RO+CD27+CD28+ and CCR7−GZM+, resembling activated T_EM_. Importantly, upon in vitro stimulation with hRSV antigen, a high proportion of CD8 T cells proliferated [33]. These results contrast with previous studies that suggest that hRSV infection impairs CD8 T cell function [103]. Further longitudinal studies in a model of hRSV adult challenge showed that hRSV-specific CD8 T cells expanded 18-fold in the blood at day 7 post-infection and decreased gradually by day 28 post-infection; at 6 months after infection, there was no difference in the frequency of CD8 T cells between pre- and post-infection levels. In contrast, in matched BAL samples, CD8 T cells increased at the same time, but were significantly more frequent than in blood (114-fold greater) and continued to rise into the convalescent period. To date, the phenotype of bRSV-specific bovine CD8 T cell has not been well defined. A single report showed that bRSV infection resulted in increased interleukin-2 receptors (CD25) expression in CD8 T cells in the lung without changes in memory markers like CD45RO, CD45R or L-selectin (CD62L) at day 10 after bRSV infection. However, in this study, phenotypic changes in T cell populations were measured in total cells isolated from lungs [30]; thus, in vitro stimulation with bRSV antigens might be necessary to detect phenotypic changes due to infection. Further longitudinal studies to analyze the phenotype of CD8 T cell responses in blood and lung during bRSV infection are warranted.

There is a poor correlation between T cell frequency and function in blood and protection against respiratory viral infections. Increasing evidence has underlined the importance of analyzing the T cells responses to vaccination and infection in the airways. Resident memory T (T_RM_) cells are a recently identified subset of non-circulating memory T cells poised to protect sites of pathogen entry. T_RM_ cells are phenotypically different from recirculating central and memory T cells. T_RM_ cells can be identified by the expression of markers such as CD69 and CD103; lack of expression of CD62L, CCR7 which promote their retention within the tissues. Although the exact origin of T_RM_ and its relationship with other circulating memory T cells is unclear, recent evidence suggests that Trm are critical for mediating resistance to respiratory viral infections [35,105,106]. Following secondary challenge from pathogens that infect the respiratory epithelium, T_RM_ cells expand and rapidly produce IFNγ [107]. Using an experimental model of human hRSV infection, Joswik et al. showed that hRSV-specific CD8 T_RM_ cell frequencies in the BAL correlated with reduced disease severity [35]. Given the prime position to respond to challenge against respiratory virus infections, an understanding of T_RM_ function and regulation is essential to promote their generation in future vaccines, for generating protective immunity. Mice and nonhuman primate models have identified CD4 and CD8 T_RM_ populations with shared characteristics with human T_RM_ cells [108,109,110]. Given the similarity in the phenotypic characteristics between the bovine and human memory T cell subsets and the parallels in the immune response between hRSV and bRSV, discussed earlier in this review, it is expected that T_RM_ cells play an important role in protection against challenge bRSV.

Numerous studies have shown that CD4 T cells and the cytokines they produce, contribute to both hRSV and bRSV immunity and disease pathogenesis. The F and G proteins are the major HLA class II restricted targets in both humans and cattle [20,111]. BRSV infection results in an increase in the activation/memory phenotype of CD4 T cells in the lungs with changes in expression of CD45R, CD45RO, CD62L and CD25, all suggesting that CD4^+^ T cells were activated during bRSV infection [30].

It is thought that a dysregulated T cell response to hRSV and bRSV contributes to immunopathology during infection. HRSV infection induces T cell responses biased away from protective Th1 cytokines, towards a dysregulated, Th2 and Th17-type cytokine response [112]. In human infants, an imbalance in the Th1 and Th2 cytokine ratio has been associated with severe hRSV infection [113,114]. Evidence of Th2 cytokine skewing also occurs during bRSV infection, as cells and lymph fluid from bRSV infected calves showed enhanced IL-4 and IL-13 production in the serum and tissues as early as day 4 post-infection, as well as increased IgE levels, eosinophilia, and lower IFNγ at 6 days post-infection [26,115,116,117,118]. These features suggest that both hRSV and bRSV might possess molecular mechanisms to enhance the production of Th2 cytokines in the host in order to prevent clearance by cytotoxic T cells and ensure replication. Further, Th2 skewing is associated with enhanced disease following FI-hRSV immunization and may be a factor in airway reactivity and wheezing common in infants with severe hRSV disease. The induction of a Th2 response by vaccination with FI-bRSV, and the induction of airway reactivity has been also demonstrated in vaccinated/infected calves [25].

Although a Th1/Th2 imbalance can explain some of the observed pathology during primary bRSV and hRSV infection, recent data in both humans and calves propose that IL-17 levels may also be detrimental during infection. We have demonstrated that calves infected with bRSV express significant levels of Th17-related cytokines, such as IL-17, IL-21 and IL-22; and both CD4 T cells and γδ T cells contribute to this response [119]. Similarly, hRSV infected infants have elevated IL-17 and Th17 promoting cytokines, IL-1β, IL-6 and IL-23 [112]. IL-17 production leads to the recruitment of neutrophils into the site of infection through induction of chemokines such as CXCL1, CCL20, IL-6 and IL-8 [120,121]. As previously discussed in this review, neutrophil infiltration contributes to the pathophysiology of both hRSV and bRSV infection. In accordance, concentrations of IL-17 and IL-8, and neutrophil counts from tracheal aspirates of newborn hRSV patients were significantly higher compared to healthy controls [122]. Current studies in our lab are underway to provide a more detailed analysis of the in vivo role of IL-17 and to increase our understanding of disease pathogenesis in bRSV.

γδ T cells are unconventional subset of the T cell population, particularly associated with mucosal epithelial surfaces. Although there is evidence that γδ T cells play a role in defense against intracellular pathogens, the extent of γδ T cell involvement in hRSV disease in humans is poorly defined. T cells expressing the γ/δ TCR are a major component of the circulating lymphocyte pool in ruminants. Studies in our lab have identified chemokine and cytokine production by bovine γδ T cells responding to *in vitro* and *in vivo* bRSV infection [123]. MCP-1, MIP-1α and IL-10 mRNA in γδ T cells increases in response to TLR3 activation [123]. Previous reports indicate that γδ T cells do not appear to contribute to the clearance of bRSV in cattle, as depletion of WC1+γδ T cells did not affect the course of bRSV infection [101]. Interestingly, peripheral γδ T cells of infants with acute hRSV infection were shown to produce significantly less IFN-γ and more IL-4 after mitogen stimulation, which was later associated with the development post-bronchiolitic wheezing during the convalescent phase [124]. In accordance, an increase in the frequency of γδ T cells producing Th-type 2 cytokines localized in BAL has been reported during allergic asthma [125]. These observations suggest that γδ T cells may play a role in the development of airway reactivity after hRSV infection.

## 9. Genetic Influence in the Host’s Immune Response to bRSV

Even in a single outbreak of human or bovine RSV disease, the clinical severity is highly variable, indicating that individual host factors or viral factors are influencing disease severity. There is growing evidence of the role of genetics in the immune responses to hRSV infection [126,127]. Janseen et al. conducted one of the most informative candidate genes studies in 384 SNPs in 220 candidate genes across different categories, in 470 human infants. Results show that susceptibility to hRSV is a complex trait, with the strongest association (both the allele and the genotype levels to the phenotype) in just a few genes involved in innate immunity, highlighting the importance of the early immune response to limit the viral infection [127]. In accordance, infants possessing single-nucleotide polymorphism (SNPs) encoding Asp299Gly and Thre3999Ile substitutions in the TLR4 domain were highly associated with hRSV symptomatic disease. Although limited, some studies have suggested that host genetics are likely to play a role in the immune response to bRSV vaccination in cattle [128,129]. Glass et al. reported two SNPs associated with the response to bRSV vaccine. The most significant SNPs was located on BTA8 which influenced IgG1 and IgG2 response and was located in TLR4 gene [128]. Supporting the role for TLR4 in host responses to bRSV.

Regions of the bovine major histocompatibility complex (BoLA) and non-MHC genes are strongly associated with the immune response to vaccination and disease resistance. Significant associations between BoLA class II DRB3 polymorphisms, particularly in the sequences encoding the peptide binding pockets, and levels of circulating antibody pre- and post-vaccination with the bRSV vaccine have been identified [128,129]. Further exploration of the role of genetics in the response to bRSV is expected to improve vaccine efficacy.

## 10. hRSV and bRSV Vaccine Development

In humans, there is currently no vaccine available. RSV vaccine development experienced a major setback in the 1960’s, with disastrous outcomes resulting from trials of a formalin-inactivated RSV vaccine. Children receiving the FI-RSV vaccine were not protected, and upon RSV infection, experienced an exaggerated clinical response compared to unvaccinated controls [130,131,132]. In one report, nearly 80% of vaccinates were hospitalized, compared to only 5% of controls [130].

Modified-live and killed vaccines have been available for bRSV infection in cattle since the early 1980’s. They are generally marketed as multivalent products that target other common respiratory pathogens. Despite their widespread availability, the continued prevalence of bRSV infection in both the beef and dairy industries leads to questions of their efficacy. In calves, there have been two reports of enhanced disease resulting after natural bRSV infection [133,134]. In both reports, bRSV-associated morbidity and mortality was significantly enhanced in immunized animals compared to non-vaccinated calves. One case occurred in a herd of 8 month old Belgian blue calves that received a beta-propiolactone inactivated vaccine [134]; and the other instance occurred in 5–7 month old calves on a beef-fattening farm in the Netherlands, following administration of a modified-live bRSV vaccine [133]. In the second case, the vaccine was administered during a natural bRSV outbreak, leading the authors to speculate that the vaccine may have enhanced disease by quantitatively or qualitatively altering host IgM and IgG responses [133]. Experimentally, vaccine-enhanced disease has been reproduced in some studies of calves vaccinated with formalin-inactivated bRSV preparations [25,27], but not in others [135,136].

Vaccine development for both calves and humans faces many of the same challenges. Specifically, the need to immunize very young target populations; to vaccinate in the face of maternal or preexisting immunity; and to induce an appropriate, robust and long-lasting immune response. Thus, the calf model is an excellent choice for RSV vaccine development, and the model has been employed for testing several encouraging vaccine candidates.

There have been recent, comprehensive reviews of candidate hRSV vaccines which are currently in preclinical and clinical trials for humans [137,138,139]. Therefore, here we focus on only a few promising candidate vaccines which have undergone testing in calves as a preclinical model, and thus may eventually benefit both species. Below, we have provided a brief summary of the strategies that are currently being employed in RSV vaccinology (also recently reviewed in [137,138,139]), followed by a more detailed description highlighting several recent candidates. Table 2 includes a summary of the vaccine candidates that are discussed below.

### 10.1. General Considerations for RSV Vaccine Development

While the main goal of hRSV and bRSV vaccine development is to induce protection against the virus, it is also important to avoid the induction of vaccine enhanced disease. Two general strategies exist for inducing protective immunity against HRSV infection in infants and young children. Direct immunization (infant and child vaccination) is ideal for activating the host’s own immune response; however, the immune system of very young infants is not well equipped to mount a robust and long-lived adaptive immune response [137,138,139,140]. Further, infant vaccination must overcome the possibility of interference from maternal antibodies. Therefore, maternal immunization is a promising strategy for protecting infants shortly after birth, which is the window of greatest susceptibility [137,138,139,140]. Maternal immunization is being actively pursued in clinical trials, as discussed below. However, maternal antibody levels wane within a few months, and maternal antibodies are not always protective against hRSV [141,142,143,144] or bRSV [89,145], thus it is likely that multiple approaches will be required to reduce the global burden of infection.

Most current hRSV vaccine candidates can be divided into 4 general categories: vector-based, subunit based, nanoparticle based and live-attenuated or chimeric vaccines [137,138,139]. Nonreplicating vaccines are safe, but have the potential to promote vaccine enhanced disease and are generally less immunogenic than replication-competent vaccines. However, nanoparticle-based platforms may overcome this defect, as they provide an antigen-depot effect which preserves and enhances antigen-presentation. Vector-based and live-attenuated vaccines can be administered intranasally, are less likely than subunit vaccines to promote enhanced disease, and have the capacity to activate both the cellular and humoral arms of the immune system. The advantages and disadvantages of these various strategies are more thoroughly reviewed elsewhere [137,138,139,140].

### 10.2. Vector-Based Vaccines

Vector-based vaccines are one promising strategy that is being investigated for both human and animal use. In recent parallel reports, a chimpanzee adenovirus-vectored vaccine candidate, PanAd3-RSV, expressing the RSV N and M2-1 proteins and a secreted form of the human RSV F protein, was tested in calves for efficacy bRSV infection [146] and for safety and immunogenicity in humans in a Phase I clinical trial [147]. The PanAd3-RSV vaccine alone, and together in a prime-boost regimen with modified vaccinia Ankara expressing the same RSV antigens (MVA-RSV), induced both cellular and humoral immune responses, and protected young seronegative calves from bRSV-associated respiratory disease [146]. In healthy adult humans, the combination of intramuscular PanAd3-RSV and intramuscular MVA-RSV was well tolerated and induced circulating F-specific IgA and IgG antibody-secreting cells, and RSV-specific, IFNγ-producing CD4 and CD8 T cells [147]. To our knowledge, the combined use of the PanAd3-RSV and MVA-RSV vectors has not advanced further in calves or clinical trials. However, a slightly modified version of the MVA vector, MVA-BN (Bavarian Nordic) RSV expressing the G protein in addition to the F, N and M2-1 proteins, is currently in Phase II development for both pediatric and elderly patients [137].

### 10.3. Live Attenuated Vaccines

Live, attenuated vaccines have been a major focus for RSV vaccine development due to the reduced likelihood of vaccine enhanced disease. Mutations and/or deletions have been made in several proteins of RSV, including NS1, NS2, SH, G, F and M2-1 to generate attenuated strains [20,140]. However, it has been difficult to select live candidates with the appropriate combination of safety and immunogenicity. Mutations and deletions in the SH gene in particular have shown some promise in the bovine and in early phase clinical trials. In independent experiments, Taylor et al. tested the efficacy of an SH gene-deleted bRSV mutant in colostrum restricted calves [74], while Blodörn et al. tested a similar gene-deletion mutant in calves with maternal antibodies [159]. In both studies, the SH-deletion vaccine was highly protective against bRSV challenge. An analogous strain of hRSV containing an SH-deletion and additional point mutations, MEDI-559, was shown safe and immunogenic in seronegative infants and children between 5–24 months of age [148]. Progress of MEDI-559 was halted due to loss of attenuating mutations that were observed in several specimens collected from clinical trials [148,149]. However, MEDI-559 was reengineered to stabilize the attenuating mutations, resulting in a new candidate strain, RSVcps2 [150]. Testing in infants and children, ages 6–24 months old, revealed the mutant to be safe, moderately immunogenic and genetically stable.

### 10.4. Subunit and Nanoparticle-Based Vaccines

The descriptions of the pre-F form of the hRSV [160] and bRSV [93] F proteins have significantly changed our understanding of RSV antigenicity and the host neutralizing antibody response. In one of the first proof-of-principal studies, mice and macaques were immunized twice with a stabilized pre-F antigen from hRSV, adjuvanted with Poly(I:C) [152]. The pre-F vaccine was highly immunogenic in both species. Monkeys developed neutralizing antibody titers to a heterologous RSV virus that were more than 80-fold higher than animals receiving a post-F-based vaccine [152]. The same group then went on to explore the immunogenicity of the pre-F formulation against BRSV infection in the neonatal calf model [93]. Young, seronegative calves received an intramuscular injection of the pre-F protein adjuvanted with oil-in-water adjuvant Montanide ISA 71G, followed by an intramuscular boost 4 weeks later. Calves receiving the stabilized, pre-F vaccine mounted a 100-fold higher neutralizing antibody response than calves receiving a similarly formulated post-F vaccine. The animals were challenged four weeks after boosting with 10^4^ plaque forming units of the Snook strain, administered via intranasal and intratracheal inoculation. Following challenge, calves receiving the pre-F vaccine had no demonstrable virus titers in the upper or lower respiratory tract, and only 1 of 5 calves developed bRSV-associated clinical signs. In contrast, post-F vaccinated calves were not significantly different from unvaccinated control calves with regards to virus shedding or clinical signs, and had intermediate levels of virus-associated lung pathology [93]. In a follow-up study to optimize adjuvant selection, seronegative calves were again immunized with the stabilized, pre-F vaccine, adjuvanted with ISA71G, as in the original publication, or with ISA71G plus Carbopol, a polyionic high molecular weight acrylic acid, mixed 1:1 [151]. Carbopol had a significant positive impact on antibody titers in rodent experiments but had no impact on the RSV neutralizing antibody titer in calves. Recently, investigators with GlaxoSmithKlein Vaccines used seropositive, adult cows as a model to study boosting of pre-existing, pre-F neutralizing responses [94], as such a strategy may be applied for maternal vaccination and for vaccination of elderly patients. In this report, a single immunization with non-adjuvanted stabilized pre-F boosted pre-existing RSV neutralizing antibody titers by 14-fold. The authors speculate that, in a model of maternal vaccination, this increase would be sufficient to result in 4–5 months of protecting neutralizing antibody titers in babies [94]. Given the many recent successes in the use of stabilized forms of the pre-F protein in preclinical testing (reviewed in [161]), and more recently, in clinical trials in human patients [153], it is doubtless that the future will bring additional investigations into the use of the pre-F in the bovine model as well.

Subunit vaccines, such as the pre-F formulations described above, are the gold standard for immunizing infants and young children due to their safety. However, the efficacy of a subunit vaccine relies heavily on a delivery vehicle that will minimize protein degradation and maximize immunogenicity. Synthetic, biodegradable particle carriers are appealing because they act as a combination adjuvant and delivery mechanisms, serving as a depot that both protects the integrity of encapsulated antigens and increases antigen availability to antigen presenting cells (recently reviewed in [162]). Poly(lactic-*co*-glycolic) acid (PLGA) is a base material that has gained considerable interest for use as a vaccine carrier. It is highly biocompatible and protects its antigen payload from degradation, thus increasing delivery efficiency [162]. Calves vaccinated with PLGA particles encapsulating the bRSV post-F and G proteins mount an IgA response in upper respiratory tract and are partially protected against bRSV challenge [154].

We have recently published our description of a novel, nanoparticle-based vaccine for use against bRSV infection in neonatal calves [89]. Polyanhydride nanoparticles are composed of biodegradable polymers of 1,6-bis (*p*-carboxyphenoxy) hexane (CPH) and 1,8-bis (*p*-carboxyphenoxy)-3,6-dioxaoctane (CPTEG). Like PLGA, polyanhydride nanoparticle-based vaccines are biocompatible and act as a combination vaccine adjuvant and delivery platform. Compared to PLGA, however, the combination CPH:CPTEG nanoparticles provide improved protection for highly labile proteins and tunable release kinetics [163,164]. The improved antigen protection is ideal for labile proteins such as the surface glycoproteins of RSV. In our recent study, we co-encapsulated recombinant post-F and G proteins from bRSV. A single, intranasal dose of vaccine was given to calves less than 4 weeks of age. The animals were then challenged four weeks after vaccination via aerosol inoculation with 10^4^ TCID_50_ BRSV strain 375. The vaccine induced robust local cellular and humoral immunity in the respiratory tract, reduced bRSV-associated pathology and viral burden, and decreased the incidence of virus shedding in neonatal calves [154].

The RSV N protein is difficult to produce as a soluble protein. However, several recombinant N proteins can be engineered together to generate 3D circulatory ring-shaped nanoparticles (N^SRS^) [165]. In calves, intramuscular administration of N^SRS^ adjuvanted with Montanide ISA71 VG, or intranasal administration of N^SRS^ adjuvanted with Montanide IMS4132 results in the induction of a robust cellular immune response, which correlates with reduced virus-associated lung pathology and reduced viral loads following bRSV challenge [155]. The N^SRS^ nanorings are capable of serving as a carrier for heterologous proteins and most recently, the nanorings were fused to the RSV FsII epitope [166], which is an epitope on the F protein which corresponds to the site recognized by the therapeutic monoclonal antibody, Palivizumab. N-induced immunity is not sterilizing, and even with a robust immune response, hosts are still permissive to RSV replication in the upper respiratory tract [155,167]. The authors speculated that the chimeric FsII-N nanorings would ideally induce N-specific cellular immunity, and an F-specific neutralizing antibody response [166]. FsII-N vaccinated mice developed significant anti-F antibody titers and were efficiently protected against RSV replication in the lungs and upper respiratory tract [166]. The mice were also spared any signs of RSV-associated disease. While further optimization is needed for desired levels of immunogenicity, the rationale is solid, and the promising early results warrant further investigations.

Like the N protein, the RSV F protein can also be assembled into 3D nanoparticle structures [168]. Novavax currently has an RSV F nanoparticle vaccine in various phases of clinical trials for pediatric, maternal and elderly cohorts. The vaccine is composed of multiple F-protein trimers, in the prefusion conformation, assembled into nanoparticle complexes and adjuvanted with aluminum hydroxide [156]. Results from clinical trials in healthy young women of childbearing age [156,158], and most recently, in elderly adults [157], suggest the formulation is well tolerated and immunogenic. Unfortunately, an earlier iteration of the F nanoparticle vaccine failed to meet its clinical endpoints in a Phase III trial in elderly adults in 2016; however, the vaccine has since been reformulated and is currently undergoing Phase III trials in infants via maternal vaccination. The results of this trial are expected to be announced in 2019.

### 10.5. The Importance of Immunization Route

While antigen and adjuvant formulations are a critical consideration in the design of efficacious vaccines, the immunization route is also of great importance for RSV. As discussed above, both IgA and IgG are important for preventing RSV infection. However, as has been shown in calves [89,145] and pediatric patients [141,142,143,144], circulating maternal antibodies are not always sufficient to protect from RSV infection and can interfere with the induction of systemic immunity. RSV-specific IgA [96,169,170] and tissue-resident CD8 T cells [35,36] have both been correlated with protection from RSV disease. Thus, we speculate that mucosal vaccination, either alone or as a heterologous strategy with parenteral vaccination, is the most promising route to pursue for use against RSV. In calves, a live-attenuated intranasal bRSV vaccine has some efficacy in animals as young as 3 days of age [171]; and our own mucosal nanoparticle vaccine was efficacious in animals as young as 3 weeks of age [89]. However, protection induced by intranasal vaccination appears to wane within a few months [172], suggesting that a heterologous vaccination strategy may be optimal. This type of regimen has been supported in other disease models [173,174,175,176,177].

## 11. Utility of the Calf Model for Testing Antiviral and Therapeutic Compounds

There are currently few options available for treating severe RSV infection in infants, and lack of appropriate animal models continues to slow preclinical development. However, recent studies have proven the calf as a relevant model for testing disease intervention strategies. Jordan et al. used the calf model to test the efficacy of a novel RSV fusion inhibitor for reducing RSV-associated disease severity [178]. When administered intravenously starting 24 or 72 h post-infection, the antiviral drug showed clear therapeutic benefit by reducing clinical disease, lung pathology and viral load. In another recent study, Walsh et al. investigated the efficacy of ibuprofen for controlling disease severity during bRSV infection in the calf [179]. NSAIDS are commonly administered to both humans and animals to control symptoms during mild respiratory infections. However, there is little evidence to support or discourage their use during RSV infection. Calves were administered ibuprofen in the milk replacer, beginning on the day of inoculation. Animals receiving ibuprofen showed a mild reduction in virus associated clinical signs, but no differences in lung pathology compared to placebo treated controls [137]. Interestingly, calves receiving ibuprofen shed higher amounts of virus, suggesting that the therapy had a negative impact on host antiviral immunity.

Neutrophil NETS are composed of extracellular DNA networked with antimicrobial peptides and histones. NET formation has recently been shown to contribute to airway obstruction during severe RSV infection [79]. In a proof-of-concept study, Cortjens et al. used aerosolized dornase alfa, a recombinant deoxyribonuclease, to target and reduce neutrophil extracellular trap formation in calves with bRSV [180]. While the treatment had some limitations, calves receiving dornase alfa showed reduced NET formation and airway obstruction in the lungs at necropsy, on day 7 post-infection. Importantly, the combined results of these studies underline the utility of the calf model as a physiologic host for testing both therapies and vaccines that may be effective against hRSV infection in human infants.

## 12. Conclusions

Development of RSV vaccines and therapeutics has been a work in progress for over 50 years. However, recent significant advancements in our understanding of RSV pathogenesis have led to an unprecedented number of promising vaccine and therapeutic candidates in the development pipeline. The bovine model has been instrumental in the advancement of several of these promising candidates. There are several important similarities in diseases pathogenesis and host antiviral immunity between hRSV and bRSV infection. If utilized appropriately, studying RSV infection in a physiologic host model such as the neonatal calf can continue to further our understanding of efficacy, selection of appropriate correlates of protection, and duration of immunity. In turn, while vaccine development for bRSV infection in cattle has been somewhat stagnant in recent years, in the true spirit of One Health, the veterinary field will ultimately benefit from advancements made in human health, as many of the same principles can be applied to improving calf health and productivity.

## Figures and Tables

**Table 1:** Samples used to evaluate immune responses to natural and experimental infection in humans and calves.

Sample	Advantages	Disadvantages	hRSV	bRSV
**Lung tissue**	Allows complete diagnosis Assessment of gross pathology and histology Viral burden Cellular immunity	Requires post-mortem species Does not represent the whole lung Need to disrupt and digest specimens in order to isolate cells	Low CD4 and CD8 T cell counts after fatal hRSV [28] Widespread neutrophil infiltration after fatal hRSV [28] Absence of NK cells [29] Eosinophils present in FI-hRSV fatal cases	CD8 T cells increased at day 3–5 p.i. [30] Viral peak at day 6 p.i. [31] Neutrophil infiltration starting at day 3 p.i. [26] Elevated mRNA levels IL-8, IL-12p40, and IFNγ at day 7 p.i. [32]
**BAL** **Pre and post mortem**	Longitudinal sampling (kinetics) Allows frequent collection Analysis of immune response at site of infection (LRT) Viral burden Cellular immunity Humoral immunity	Requires trained staff and specific materials Invasive A histopathological study cannot be performed Does not represent the whole lung Dilution of secretions too high to preform humoral analysis ex vivo	Increased neutrophil [85% of total cell counts) [33,34]] Virus-specific CD8 T cells increased at day 10 p.i. [33,35] Viral peak at day 3–6 p.i. IL-8, MIP-1α, MCP-1 and RANTES, IFNγ, IL-4, IL-5, IL-10, IL-9, IL-17A, IL-12, IL-1β, IP-10 [36] IL-6 and TNFα increased with bronchitis [37]	Increased neutrophil peak at day 6–8 p.i. [38] CD8 T cells increased at day 10–15 p.i. [30,32] TNFα, IL-6, IL-8 at day 3 p.i. [39] TNFα and IFNγ peak around day 6 p.i. [26,31] Viral peak at day 6 p.i. [38,39] IgM and IgA detected at 8–10 days p.i. [40] Eosinophils increased at day 4 p.i. (FI-bRSV vaccinates) compared to day 9 p.i. non-vaccinated [25]
**Nasal fluid**	Longitudinal sampling (kinetics) Non-invasive Allows frequent collection Analysis of immune response at site of infection (URT) Viral burden Cellular immunity Humoral immunity	Representation of the upper respiratory tract only	Increased IL-8, RANTES, IL-6 and TNFα [41] Viral load peaks around days 6–8 p.i. [35] Increased IgG and IgA [42] IFNγ, IL-12, IL-1β, MCP-1, MCP-1α, MCP-1β, IP-10 [36]	IgM and IgA detected at day 8–10 p.i [40] Viral load peaks around day 8 p.i. [40]
**Nasopharyngeal or tracheal aspirate**	Viral load Cellular immunity Humoral immunity	Requires trained staff and specific materials Representation of the upper respiratory tract only	CD8 T cells peak 10 days after onset of symptoms [43] Increased neutrophils MIP-1α and RANTES elevated with severe disease [44] IgE elevated in patients with bronchiolitis	Viral burden peaks 5–7 days p.i. [26,30] Elevated CD8 T cells at day 10 p.i. [30]
**Peripheral blood**	Easy to perform Non-invasive Allows frequent collection Longitudinal sampling (kinetics) Cellular immunity Humoral immunity	Representation of the peripheral response may underestimate the local response	CD8 T cell maximal peak at day 11–15 after onset of the primary symptoms Increased IL-8, IL-6, TNFα [45,46] IgM peaks during the first week p.i. IgG peaks during the second week p.i. High IgE in severe hRSV IFNγ, IL-12, IL-4, IL-10, IP-10 [36]	IgM and IgA detected at day 8–10 p.i. [40] IgG1 elevated at day 13–17 p.i. IgG2 detected at 1–3 months p.i. IgE elevated at day 9 p.i. (FI-bRSV vaccinates) [25] IgM and IgG1 titers elevated about 6–10 days p.i. IgG2 detected around 3 weeks p.i.

**Table 2:** Overview of vaccine candidates which have undergone preclinical testing in calves.

Vaccine Candidate	Category	Antigen/Adjuvant	Host Species and Current Testing Phase ^a^	Immunization Route	References
PanAd3-RSV prime/MVA-RSV **boost**	Vector	N, M2, F; no adjuvant	bovine	IM ^b^/IM or IN ^c^/IM	[146]
		N, M2, F; no adjuvant	Human (Phase I)	IM/IM or IN/IM	[147]
MVA-BN	Vector	F, G, N, M2; no adjuvant	Human (Phase II)	IM	[137]
SH gene deletion	Live-attenuated	All native genes except SH	bovine	IN/IT ^d^ IN	[74] [39]
		Medi559: Gene-deletion for SH plus additional point mutations	Human (Phase IIa)	IN	[148,149]
		RSVcps2: Similar to Medi559 with additional stabilizing mutations	Human (Phase I)	IN	[150]
Pre-F	Subunit	Pre-F with Montanide ISA71 adjuvant	bovine	IM	[93,151]
		Pre-F, no adjuvant	bovine	IM	[94]
		Pre-F with Poly(I:C) adjuvant	Macaques	IM	[152]
		Pre-F with alum	Human (Phase II)	IM	[153]
PLGA encapsulating **post-F and G**	Nanoparticle	Post-F and G encapsulated in PLGA, no additional adjuvant	bovine	IN	[154]
BRSV-F/G Nanovaccine	Nanoparticle	Post-F and G encapsulated in CPH:CPTEG particle, no additional adjuvant	bovine	IN	[89]
N nanorings (N^SRS^)	Nanoparticle	N^SRS^ with Montanide ISA71 adjuvant	bovine	IM	[155]
		N^SRS^ with Montanide IMS4132 adjuvant	bovine	IN	[155]
RSV F nanoparticle	Nanoparticle	Near-full-length F (pre-F conformation) with aluminum hydroxide	Human (Phase III: infants via maternal immunization)	IM	[156,157,158]

^a^ Preclinical testing in rodents is not captured. ^b^ IM: intramuscular; ^c^ IN: intranasal; ^d^ intratracheal.

## References

[1] Lozano R., Naghavi M., Foreman K., Lim S., Shibuya K., Aboyans V., Abraham J., Adair T., Aggarwal R., Ahn S.Y. (2012). Global and regional mortality from 235 causes of death for 20 age groups in 1990 and 2010: A systematic analysis for the Global Burden of Disease Study 2010. Lancet.

[2] Nair H., Nokes D.J., Gessner B.D., Dherani M., Madhi S.A., Singleton R.J., O’Brien K.L., Roca A., Wright P.F., Bruce N. (2010). Global burden of acute lower respiratory infections due to respiratory syncytial virus in young children: A systematic review and meta-analysis. Lancet.

[3] Glezen W.P., Taber L.H., Frank A.L., Kasel J.A. (1986). Risk of primary infection and reinfection with respiratory syncytial virus. Am. J. Dis. Child.

[4] Falsey A.R. (2005). Respiratory syncytial virus infection in elderly and high-risk adults. Exp. Lung Res..

[5] Johnson K.K., Pendell D.L. (2017). Market Impacts of Reducing the Prevalence of Bovine Respiratory Disease in United States Beef Cattle Feedlots. Front. Vet. Sci..

[6] Falsey A.R. (2007). Respiratory syncytial virus infection in adults. Semin. Respir. Crit. Care Med..

[7] Valarcher J.F., Schelcher F., Bourhy H. (2000). Evolution of bovine respiratory syncytial virus. J. Virol..

[8] Van der Poel W.H., Brand A., Kramps J.A., Van Oirschot J.T. (1994). Respiratory syncytial virus infections in human beings and in cattle. J. Infect..

[9] Schmidt M.E., Varga S.M. (2017). Modulation of the host immune response by respiratory syncytial virus proteins. J. Microbiol..

[10] Anderson L.J., Hierholzer J.C., Tsou C., Hendry R.M., Fernie B.F., Stone Y., McIntosh K. (1985). Antigenic characterization of respiratory syncytial virus strains with monoclonal antibodies. J. Infect. Dis..

[11] Cristina J., Lopez J.A., Albo C., Garcia-Barreno B., Garcia J., Melero J.A., Portela A. (1990). Analysis of genetic variability in human respiratory syncytial virus by the RNase A mismatch cleavage method: Subtype divergence and heterogeneity. Virology.

[12] Furze J.M., Roberts S.R., Wertz G.W., Taylor G. (1997). Antigenically distinct G glycoproteins of BRSV strains share a high degree of genetic homogeneity. Virology.

[13] Deplanche M., Lemaire M., Mirandette C., Bonnet M., Schelcher F., Meyer G. (2007). In vivo evidence for quasispecies distributions in the bovine respiratory syncytial virus genome. J. Gen. Virol..

[14] McLellan J.S., Yang Y., Graham B.S., Kwong P.D. (2011). Structure of respiratory syncytial virus fusion glycoprotein in the postfusion conformation reveals preservation of neutralizing epitopes. J. Virol..

[15] Johnson P.R., Spriggs M.K., Olmsted R.A., Collins P.L. (1987). The G glycoprotein of human respiratory syncytial viruses of subgroups A and B: Extensive sequence divergence between antigenically related proteins. Proc. Natl. Acad. Sci. USA.

[16] Garcia O., Martin M., Dopazo J., Arbiza J., Frabasile S., Russi J., Hortal M., Perez-Brena P., Martinez I., Garcia-Barreno B. (1994). Evolutionary pattern of human respiratory syncytial virus (subgroup A): Cocirculating lineages and correlation of genetic and antigenic changes in the G glycoprotein. J. Virol..

[17] Woelk C.H., Holmes E.C. (2001). Variable immune-driven natural selection in the attachment (G) glycoprotein of respiratory syncytial virus (RSV). J. Mol. Evol..

[18] Prozzi D., Walravens K., Langedijk J.P., Daus F., Kramps J.A., Letesson J.J. (1997). Antigenic and molecular analyses of the variability of bovine respiratory syncytial virus G glycoprotein. J. Gen. Virol..

[19] Lukens M.V., Claassen E.A., de Graaff P.M., van Dijk M.E., Hoogerhout P., Toebes M., Schumacher T.N., van der Most R.G., Kimpen J.L., van Bleek G.M. (2006). Characterization of the CD8+ T cell responses directed against respiratory syncytial virus during primary and secondary infection in C57BL/6 mice. Virology.

[20] Meyer G., Deplanche M., Schelcher F. (2008). Human and bovine respiratory syncytial virus vaccine research and development. Comp. Immunol. Microbiol. Infect. Dis..

[21] Griffiths C., Drews S.J., Marchant D.J. (2017). Respiratory Syncytial Virus: Infection, Detection, and New Options for Prevention and Treatment. Clin. Microbiol. Rev..

[22] Collins P.L., Graham B.S. (2008). Viral and host factors in human respiratory syncytial virus pathogenesis. J. Virol..

[23] Hall C.B., Simőes E.A., Anderson L.J. (2013). Clinical and epidemiologic features of respiratory syncytial virus. Curr. Top. Microbiol. Immunol..

[24] Sacco R.E., McGill J.L., Pillatzki A.E., Palmer M.V., Ackermann M.R. (2014). Respiratory syncytial virus infection in cattle. Vet. Pathol..

[25] Antonis A.F., Schrijver R.S., Daus F., Steverink P.J., Stockhofe N., Hensen E.J., Langedijk J.P., van der Most R.G. (2003). Vaccine-induced immunopathology during bovine respiratory syncytial virus infection: Exploring the parameters of pathogenesis. J. Virol..

[26] Antonis A.F., de Jong M.C., van der Poel W.H., van der Most R.G., Stockhofe-Zurwieden N., Kimman T., Schrijver R.S. (2010). Age-dependent differences in the pathogenesis of bovine respiratory syncytial virus infections related to the development of natural immunocompetence. J. Gen. Virol..

[27] Gershwin L.J., Schelegle E.S., Gunther R.A., Anderson M.L., Woolums A.R., Larochelle D.R., Boyle G.A., Friebertshauser K.E., Singer R.S. (1998). A bovine model of vaccine enhanced respiratory syncytial virus pathophysiology. Vaccine.

[28] Welliver T.P., Garofalo R.P., Hosakote Y., Hintz K.H., Avendano L., Sanchez K., Velozo L., Jafri H., Chavez-Bueno S., Ogra P.L. (2007). Severe human lower respiratory tract illness caused by respiratory syncytial virus and influenza virus is characterized by the absence of pulmonary cytotoxic lymphocyte responses. J. Infect. Dis..

[29] Welliver T.P., Reed J.L., Welliver R.C. (2008). Respiratory syncytial virus and influenza virus infections: Observations from tissues of fatal infant cases. Pediatr. Infect. Dis. J..

[30] Mcinnes E., Sopp P., Howard C.J., Taylor G. (1999). Phenotypic analysis of local cellular responses in calves infected with bovine respiratory syncytial virus. Immunology.

[31] Røntved C.M., Tjørnehøj K., Viuff B., Larsen L.E., Godson D.L., Rønsholt L., Alexandersen S. (2000). Increased pulmonary secretion of tumor necrosis factor-alpha in calves experimentally infected with bovine respiratory syncytial virus. Vet. Immunol. Immunopathol..

[32] Sacco R.E., Nonnecke B.J., Palmer M.V., Waters W.R., Lippolis J.D., Reinhardt T.A. (2012). Differential expression of cytokines in response to respiratory syncytial virus infection of calves with high or low circulating 25-hydroxyvitamin D3. PLoS ONE.

[33] Heidema J., Lukens M.V., van Maren W.W., van Dijk M.E., Otten H.G., van Vught A.J., van der Werff D.B., van Gestel S.J., Semple M.G., Smyth R.L. (2007). CD8+ T cell responses in bronchoalveolar lavage fluid and peripheral blood mononuclear cells of infants with severe primary respiratory syncytial virus infections. J. Immunol..

[34] McNamara P.S., Ritson P., Selby A., Hart C.A., Smyth R.L. (2003). Bronchoalveolar lavage cellularity in infants with severe respiratory syncytial virus bronchiolitis. Arch. Dis. Child.

[35] Jozwik A., Habibi M.S., Paras A., Zhu J., Guvenel A., Dhariwal J., Almond M., Wong E.H., Sykes A., Maybeno M. (2015). RSV-specific airway resident memory CD8+ T cells and differential disease severity after experimental human infection. Nat. Commun..

[36] Russell C.D., Unger S.A., Walton M., Schwarze J. (2017). The Human Immune Response to Respiratory Syncytial Virus Infection. Clin. Microbiol. Rev..

[37] McNamara P.S., Flanagan B.F., Selby A.M., Hart C.A., Smyth R.L. (2004). Pro- and anti-inflammatory responses in respiratory syncytial virus bronchiolitis. Eur. Respir. J..

[38] Taylor G., Thomas L.H., Stott E.J. (1989). Effect of vaccination on cell populations in lung washes from calves after infection with respiratory syncytial virus. Res. Vet. Sci..

[39] Blodörn K., Hägglund S., Gavier-Widen D., Eléouët J.F., Riffault S., Pringle J., Taylor G., Valarcher J.F. (2015). A bovine respiratory syncytial virus model with high clinical expression in calves with specific passive immunity. BMC Vet. Res..

[40] Kimman T.G., Westenbrink F., Schreuder B.E., Straver P.J. (1987). Local and systemic antibody response to bovine respiratory syncytial virus infection and reinfection in calves with and without maternal antibodies. J. Clin. Microbiol..

[41] Laham F.R., Israele V., Casellas J.M., Garcia A.M., Lac Prugent C.M., Hoffman S.J., Hauer D., Thumar B., Name M.I., Pascual A. (2004). Differential production of inflammatory cytokines in primary infection with human metapneumovirus and with other common respiratory viruses of infancy. J. Infect. Dis..

[42] Murphy B.R., Graham B.S., Prince G.A., Walsh E.E., Chanock R.M., Karzon D.T., Wright P.F. (1986). Serum and nasal-wash immunoglobulin G and A antibody response of infants and children to respiratory syncytial virus F and G glycoproteins following primary infection. J. Clin. Microbiol..

[43] Heidema J., Rossen J.W., Lukens M.V., Ketel M.S., Scheltens E., Kranendonk M.E., van Maren W.W., van Loon A.M., Otten H.G., Kimpen J.L. (2008). Dynamics of human respiratory virus-specific CD8+ T cell responses in blood and airways during episodes of common cold. J. Immunol..

[44] Sheeran P., Jafri H., Carubelli C., Saavedra J., Johnson C., Krisher K., Sanchez P.J., Ramilo O. (1999). Elevated cytokine concentrations in the nasopharyngeal and tracheal secretions of children with respiratory syncytial virus disease. Pediatr. Infect. Dis. J..

[45] Bont L., Heijnen C.J., Kavelaars A., van Aalderen W.M., Brus F., Draaisma J.T., Geelen S.M., van Vught H.J., Kimpen J.L. (1999). Peripheral blood cytokine responses and disease severity in respiratory syncytial virus bronchiolitis. Eur. Respir. J..

[46] Hornsleth A., Klug B., Nir M., Johansen J., Hansen K.S., Christensen L.S., Larsen L.B. (1998). Severity of respiratory syncytial virus disease related to type and genotype of virus and to cytokine values in nasopharyngeal secretions. Pediatr. Infect. Dis. J..

[47] Fach S.J., Meyerholz D.K., Gallup J.M., Ackermann M.R., Lehmkuhl H.D., Sacco R.E. (2007). Neonatal ovine pulmonary dendritic cells support bovine respiratory syncytial virus replication with enhanced interleukin (IL)-4 And IL-10 gene transcripts. Viral Immunol..

[48] Meyerholz D.K., Grubor B., Fach S.J., Sacco R.E., Lehmkuhl H.D., Gallup J.M., Ackermann M.R. (2004). Reduced clearance of respiratory syncytial virus infection in a preterm lamb model. Microbes Infect. Inst. Pasteur.

[49] Woolums A.R., Anderson M.L., Gunther R.A., Schelegle E.S., LaRochelle D.R., Singer R.S., Boyle G.A., Friebertshauser K.E., Gershwin L.J. (1999). Evaluation of severe disease induced by aerosol inoculation of calves with bovine respiratory syncytial virus. Am. J. Vet. Res..

[50] Gershwin L.J. (2007). Bovine respiratory syncytial virus infection: Immunopathogenic mechanisms. Anim. Health Res. Rev..

[51] Caswell J.L., Williams K.J., Maxie M.G. (2008). Respiratory System. Jubb, Kennedy, and Palmer’s Pathology of Domestic Animals.

[52] Kimman T.G., Straver P.J., Zimmer G.M. (1989). Pathogenesis of naturally acquired bovine respiratory syncytial virus infection in calves: Morphologic and serologic findings. Am. J. Vet. Res..

[53] Aherne W., Bird T., Court S.D., Gardner P.S., McQuillin J. (1970). Pathological changes in virus infections of the lower respiratory tract in children. J. Clin. Pathol..

[54] Bem R.A., Domachowske J.B., Rosenberg H.F. (2011). Animal models of human respiratory syncytial virus disease. Am. J. Physiol. Lung Cell. Mol. Physiol..

[55] Johnson J.E., Gonzales R.A., Olson S.J., Wright P.F., Graham B.S. (2007). The histopathology of fatal untreated human respiratory syncytial virus infection. Mod. Pathol..

[56] Viuff B., Tjornehoj K., Larsen L.E., Rontved C.M., Uttenthal A., Ronsholt L., Alexandersen S. (2002). Replication and clearance of respiratory syncytial virus: Apoptosis is an important pathway of virus clearance after experimental infection with bovine respiratory syncytial virus. Am. J. Pathol..

[57] Kim T.H., Lee H.K. (2014). Innate immune recognition of respiratory syncytial virus infection. BMB Rep..

[58] Valarcher J.F., Taylor G. (2007). Bovine respiratory syncytial virus infection. Vet. Res..

[59] Werling D., Koss M., Howard C.J., Taylor G., Langhans W., Hope J.C. (2002). Role of bovine chemokines produced by dendritic cells in respiratory syncytial virus-induced T cell proliferation. Vet. Immunol. Immunopathol..

[60] Lukens M.V., van de Pol A.C., Coenjaerts F.E., Jansen N.J., Kamp V.M., Kimpen J.L., Rossen J.W., Ulfman L.H., Tacke C.E., Viveen M.C. (2010). A systemic neutrophil response precedes robust CD8(+) T-cell activation during natural respiratory syncytial virus infection in infants. J. Virol..

[61] Smyth R.L., Mobbs K.J., O’Hea U., Ashby D., Hart C.A. (2002). Respiratory syncytial virus bronchiolitis: Disease severity, interleukin-8, and virus genotype. Pediatr. Pulmonol..

[62] Haeberle H.A., Takizawa R., Casola A., Brasier A.R., Dieterich H.J., Van Rooijen N., Gatalica Z., Garofalo R.P. (2002). Respiratory syncytial virus-induced activation of nuclear factor-kappaB in the lung involves alveolar macrophages and toll-like receptor 4-dependent pathways. J. Infect. Dis..

[63] Lizundia R., Sauter K.S., Taylor G., Werling D. (2008). Host species-specific usage of the TLR4-LPS receptor complex. Innate Immun..

[64] Scagnolari C., Midulla F., Pierangeli A., Moretti C., Bonci E., Berardi R., De Angelis D., Selvaggi C., Di Marco P., Girardi E. (2009). Gene expression of nucleic acid-sensing pattern recognition receptors in children hospitalized for respiratory syncytial virus-associated acute bronchiolitis. Clin. Vaccine Immunol..

[65] Sabbah A., Chang T.H., Harnack R., Frohlich V., Tominaga K., Dube P.H., Xiang Y., Bose S. (2009). Activation of innate immune antiviral responses by Nod2. Nat. Immunol..

[66] Liu P., Jamaluddin M., Li K., Garofalo R.P., Casola A., Brasier A.R. (2007). Retinoic acid-inducible gene I mediates early antiviral response and Toll-like receptor 3 expression in respiratory syncytial virus-infected airway epithelial cells. J. Virol..

[67] Marr N., Wang T.I., Kam S.H., Hu Y.S., Sharma A.A., Lam A., Markowski J., Solimano A., Lavoie P.M., Turvey S.E. (2014). Attenuation of respiratory syncytial virus-induced and RIG-I-dependent type I IFN responses in human neonates and very young children. J. Immunol..

[68] Bossert B., Conzelmann K.K. (2002). Respiratory syncytial virus (RSV) nonstructural (NS) proteins as host range determinants: A chimeric bovine RSV with NS genes from human RSV is attenuated in interferon-competent bovine cells. J. Virol..

[69] Schlender J., Bossert B., Buchholz U., Conzelmann K.K. (2000). Bovine respiratory syncytial virus nonstructural proteins NS1 and NS2 cooperatively antagonize alpha/beta interferon-induced antiviral response. J. Virol..

[70] Lo M.S., Brazas R.M., Holtzman M.J. (2005). Respiratory syncytial virus nonstructural proteins NS1 and NS2 mediate inhibition of Stat2 expression and alpha/beta interferon responsiveness. J. Virol..

[71] Spann K.M., Tran K.C., Collins P.L. (2005). Effects of nonstructural proteins NS1 and NS2 of human respiratory syncytial virus on interferon regulatory factor 3, NF-kappaB, and proinflammatory cytokines. J. Virol..

[72] Bossert B., Marozin S., Conzelmann K.K. (2003). Nonstructural proteins NS1 and NS2 of bovine respiratory syncytial virus block activation of interferon regulatory factor 3. J. Virol..

[73] Valarcher J.F., Furze J., Wyld S., Cook R., Conzelmann K.K., Taylor G. (2003). Role of alpha/beta interferons in the attenuation and immunogenicity of recombinant bovine respiratory syncytial viruses lacking NS proteins. J. Virol..

[74] Taylor G., Wyld S., Valarcher J.F., Guzman E., Thom M., Widdison S., Buchholz U.J. (2014). Recombinant bovine respiratory syncytial virus with deletion of the SH gene induces increased apoptosis and pro-inflammatory cytokines in vitro, and is attenuated and induces protective immunity in calves. J. Gen. Virol..

[75] Whitehead S.S., Bukreyev A., Teng M.N., Firestone C.Y., St Claire M., Elkins W.R., Collins P.L., Murphy B.R. (1999). Recombinant respiratory syncytial virus bearing a deletion of either the NS2 or SH gene is attenuated in chimpanzees. J. Virol..

[76] Pollock N., Taylor G., Jobe F., Guzman E. (2017). Modulation of the transcription factor NF-kappaB in antigen-presenting cells by bovine respiratory syncytial virus small hydrophobic protein. J. Gen. Virol..

[77] Hagglund S., Blodorn K., Naslund K., Vargmar K., Lind S.B., Mi J., Arainga M., Riffault S., Taylor G., Pringle J. (2017). Proteome analysis of bronchoalveolar lavage from calves infected with bovine respiratory syncytial virus-Insights in pathogenesis and perspectives for new treatments. PLoS ONE.

[78] Funchal G.A., Jaeger N., Czepielewski R.S., Machado M.S., Muraro S.P., Stein R.T., Bonorino C.B., Porto B.N. (2015). Respiratory syncytial virus fusion protein promotes TLR-4-dependent neutrophil extracellular trap formation by human neutrophils. PLoS ONE.

[79] Cortjens B., de Boer O.J., de Jong R., Antonis A.F., Sabogal Pineros Y.S., Lutter R., van Woensel J.B., Bem R.A. (2016). Neutrophil extracellular traps cause airway obstruction during respiratory syncytial virus disease. J. Pathol..

[80] Sigurs N., Aljassim F., Kjellman B., Robinson P.D., Sigurbergsson F., Bjarnason R., Gustafsson P.M. (2010). Asthma and allergy patterns over 18 years after severe RSV bronchiolitis in the first year of life. Thorax.

[81] (1998). Palivizumab, a humanized respiratory syncytial virus monoclonal antibody, reduces hospitalization from respiratory syncytial virus infection in high-risk infants. The IMpact-RSV Study Group. Pediatrics.

[82] Kimman T.G., Westenbrink F. (1990). Immunity to human and bovine respiratory syncytial virus. Arch. Virol..

[83] Vissers M., Ahout I.M., de Jonge M.I., Ferwerda G. (2015). Mucosal IgG Levels Correlate Better with Respiratory Syncytial Virus Load and Inflammation than Plasma IgG Levels. Clin. Vaccine Immunol..

[84] Crowe J.E., Williams J.V. (2003). Immunology of viral respiratory tract infection in infancy. Paediatr. Respir. Rev..

[85] Taylor G., Thomas L.H., Furze J.M., Cook R.S., Wyld S.G., Lerch R., Hardy R., Wertz G.W. (1997). Recombinant vaccinia viruses expressing the F, G or N, but not the M2, protein of bovine respiratory syncytial virus (BRSV) induce resistance to BRSV challenge in the calf and protect against the development of pneumonic lesions. J. Gen. Virol..

[86] Jorquera P.A., Choi Y., Oakley K.E., Powell T.J., Boyd J.G., Palath N., Haynes L.M., Anderson L.J., Tripp R.A. (2013). Nanoparticle vaccines encompassing the respiratory syncytial virus (RSV) G protein CX3C chemokine motif induce robust immunity protecting from challenge and disease. PLoS ONE.

[87] Schrijver R.S., Langedijk J.P., Keil G.M., Middel W.G., Maris-Veldhuis M., Van Oirschot J.T., Rijsewijk F.A. (1997). Immunization of cattle with a BHV1 vector vaccine or a DNA vaccine both coding for the G protein of BRSV. Vaccine.

[88] Taylor G., Rijsewijk F.A., Thomas L.H., Wyld S.G., Gaddum R.M., Cook R.S., Morrison W.I., Hensen E., van Oirschot J.T., Keil G. (1998). Resistance to bovine respiratory syncytial virus (BRSV) induced in calves by a recombinant bovine herpesvirus-1 expressing the attachment glycoprotein of BRSV. J. Gen. Virol..

[89] McGill J.L., Kelly S.M., Kumar P., Speckhart S., Haughney S.L., Henningson J., Narasimhan B., Sacco R.E. (2018). Efficacy of mucosal polyanhydride nanovaccine against respiratory syncytial virus infection in the neonatal calf. Sci. Rep..

[90] Swanson K.A., Settembre E.C., Shaw C.A., Dey A.K., Rappuoli R., Mandl C.W., Dormitzer P.R., Carfi A. (2011). Structural basis for immunization with postfusion respiratory syncytial virus fusion F glycoprotein (RSV F) to elicit high neutralizing antibody titers. Proc. Natl. Acad. Sci. USA.

[91] Raghunandan R., Lu H., Zhou B., Xabier M.G., Massare M.J., Flyer D.C., Fries L.F., Smith G.E., Glenn G.M. (2014). An insect cell derived respiratory syncytial virus (RSV) F nanoparticle vaccine induces antigenic site II antibodies and protects against RSV challenge in cotton rats by active and passive immunization. Vaccine.

[92] Ngwuta J.O., Chen M., Modjarrad K., Joyce M.G., Kanekiyo M., Kumar A., Yassine H.M., Moin S.M., Killikelly A.M., Chuang G.Y. (2015). Prefusion F-specific antibodies determine the magnitude of RSV neutralizing activity in human sera. Sci. Transl. Med..

[93] Zhang B., Chen L., Silacci C., Thom M., Boyington J.C., Druz A., Joyce M.G., Guzman E., Kong W.P., Lai Y.T. (2017). Protection of calves by a prefusion-stabilized bovine RSV F vaccine. NPJ Vaccines.

[94] Steff A.M., Monroe J., Friedrich K., Chandramouli S., Nguyen T.L., Tian S., Vandepaer S., Toussaint J.F., Carfi A. (2017). Pre-fusion RSV F strongly boosts pre-fusion specific neutralizing responses in cattle pre-exposed to bovine RSV. Nat. Commun..

[95] Boxus M., Tignon M., Roels S., Toussaint J.F., Walravens K., Benoit M.A., Coppe P., Letesson J.J., Letellier C., Kerkhofs P. (2007). DNA immunization with plasmids encoding fusion and nucleocapsid proteins of bovine respiratory syncytial virus induces a strong cell-mediated immunity and protects calves against challenge. J. Virol..

[96] Habibi M.S., Jozwik A., Makris S., Dunning J., Paras A., DeVincenzo J.P., de Haan C.A., Wrammert J., Openshaw P.J., Chiu C. (2015). Impaired Antibody-mediated Protection and Defective IgA B-Cell Memory in Experimental Infection of Adults with Respiratory Syncytial Virus. Am. J. Respir. Crit. Care Med..

[97] Kalina W.V., Woolums A.R., Gershwin L.J. (2005). Formalin-inactivated bovine RSV vaccine influences antibody levels in bronchoalveolar lavage fluid and disease outcome in experimentally infected calves. Vaccine.

[98] Wagner D.K., Muelenaer P., Henderson F.W., Snyder M.H., Reimer C.B., Walsh E.E., Anderson L.J., Nelson D.L., Murphy B.R. (1989). Serum immunoglobulin G antibody subclass response to respiratory syncytial virus F and G glycoproteins after first, second, and third infections. J. Clin. Microbiol..

[99] Antonis A.F., Claassen E.A., Hensen E.J., de Groot R.J., de Groot-Mijnes J.D., Schrijver R.S., van der Most R.G. (2006). Kinetics of antiviral CD8 T cell responses during primary and post-vaccination secondary bovine respiratory syncytial virus infection. Vaccine.

[100] Gaddum R.M., Cook R.S., Furze J.M., Ellis S.A., Taylor G. (2003). Recognition of bovine respiratory syncytial virus proteins by bovine CD8+ T lymphocytes. Immunology.

[101] Taylor G., Thomas L.H., Wyld S.G., Furze J., Sopp P., Howard C.J. (1995). Role of T-lymphocyte subsets in recovery from respiratory syncytial virus infection in calves. J. Virol..

[102] West K., Petrie L., Konoby C., Haines D.M., Cortese V., Ellis J.A. (1999). The efficacy of modified-live bovine respiratory syncytial virus vaccines in experimentally infected calves. Vaccine.

[103] Gaddum R.M., Cook R.S., Thomas L.H., Taylor G. (1996). Primary cytotoxic T-cell responses to bovine respiratory syncytial virus in calves. Immunology.

[104] Openshaw P.J., Chiu C. (2013). Protective and dysregulated T cell immunity in RSV infection. Curr. Opin. Virol..

[105] Turner D.L., Bickham K.L., Thome J.J., Kim C.Y., D’Ovidio F., Wherry E.J., Farber D.L. (2014). Lung niches for the generation and maintenance of tissue-resident memory T cells. Mucosal Immunol..

[106] Teijaro J.R., Turner D., Pham Q., Wherry E.J., Lefrancois L., Farber D.L. (2011). Cutting edge: Tissue-retentive lung memory CD4 T cells mediate optimal protection to respiratory virus infection. J. Immunol..

[107] Wu T., Hu Y., Lee Y.T., Bouchard K.R., Benechet A., Khanna K., Cauley L.S. (2014). Lung-resident memory CD8 T cells (TRM) are indispensable for optimal cross-protection against pulmonary virus infection. J. Leukoc. Biol..

[108] Zens K.D., Chen J.K., Farber D.L. (2016). Vaccine-generated lung tissue-resident memory T cells provide heterosubtypic protection to influenza infection. JCI Insight.

[109] Kumar B.V., Ma W., Miron M., Granot T., Guyer R.S., Carpenter D.J., Senda T., Sun X., Ho S.H., Lerner H. (2017). Human Tissue-Resident Memory T Cells Are Defined by Core Transcriptional and Functional Signatures in Lymphoid and Mucosal Sites. Cell Rep..

[110] Pichyangkul S., Yongvanitchit K., Limsalakpetch A., Kum-Arb U., Im-Erbsin R., Boonnak K., Thitithayanont A., Jongkaewwattana A., Wiboon-ut S., Mongkolsirichaikul D. (2015). Tissue Distribution of Memory T and B Cells in Rhesus Monkeys following Influenza A Infection. J. Immunol..

[111] Fogg M.H., Parsons K.R., Thomas L.H., Taylor G. (2001). Identification of CD4+ T cell epitopes on the fusion (F) and attachment (G) proteins of bovine respiratory syncytial virus (BRSV). Vaccine.

[112] Lambert L., Sagfors A.M., Openshaw P.J., Culley F.J. (2014). Immunity to RSV in Early-Life. Front. Immunol..

[113] Legg J.P., Hussain I.R., Warner J.A., Johnston S.L., Warner J.O. (2003). Type 1 and type 2 cytokine imbalance in acute respiratory syncytial virus bronchiolitis. Am. J. Respir. Crit. Care Med..

[114] Kristjansson S., Bjarnarson S.P., Wennergren G., Palsdottir A.H., Arnadottir T., Haraldsson A., Jonsdottir I. (2005). Respiratory syncytial virus and other respiratory viruses during the first 3 months of life promote a local TH2-like response. J. Allergy Clin. Immunol..

[115] Miao C., Woolums A.R., Zarlenga D.S., Brown C.C., Brown J.C., Williams S.M., Scott M.A. (2004). Effects of a single intranasal dose of modified-live bovine respiratory syncytial virus vaccine on cytokine messenger RNA expression following viral challenge in calves. Am. J. Vet. Res..

[116] Stewart R.S., Gershwin L.J. (1989). Detection of IgE antibodies to bovine respiratory syncytial virus. Vet. Immunol. Immunopathol..

[117] Stewart R.S., Gershwin L.J. (1989). Role of IgE in the pathogenesis of bovine respiratory syncytial virus in sequential infections in vaccinated and nonvaccinated calves. Am. J. Vet. Res..

[118] Green C.A., Sande C.J., de Lara C., Thompson A.J., Silva-Reyes L., Napolitano F., Pierantoni A., Capone S., Vitelli A., Klenerman P. (2018). Humoral and cellular immunity to RSV in infants, children and adults. Vaccine.

[119] McGill J.L., Rusk R.A., Guerra-Maupome M., Briggs R.E., Sacco R.E. (2016). Bovine Gamma Delta T Cells Contribute to Exacerbated IL-17 Production in Response to Co-Infection with Bovine RSV and Mannheimia haemolytica. PLoS ONE.

[120] Onishi R.M., Gaffen S.L. (2010). Interleukin-17 and its target genes: Mechanisms of interleukin-17 function in disease. Immunology.

[121] Khader S.A., Gaffen S.L., Kolls J.K. (2009). Th17 cells at the crossroads of innate and adaptive immunity against infectious diseases at the mucosa. Mucosal Immunol..

[122] Stoppelenburg A.J., Salimi V., Hennus M., Plantinga M., Huis in ’t Veld R., Walk J., Meerding J., Coenjaerts F., Bont L., Boes M. (2013). Local IL-17A potentiates early neutrophil recruitment to the respiratory tract during severe RSV infection. PLoS ONE.

[123] McGill J.L., Nonnecke B.J., Lippolis J.D., Reinhardt T.A., Sacco R.E. (2013). Differential chemokine and cytokine production by neonatal bovine gammadelta T-cell subsets in response to viral toll-like receptor agonists and in vivo respiratory syncytial virus infection. Immunology.

[124] Aoyagi M., Shimojo N., Sekine K., Nishimuta T., Kohno Y. (2003). Respiratory syncytial virus infection suppresses IFN-gamma production of gammadelta T cells. Clin. Exp. Immunol..

[125] Girardi M., Oppenheim D.E., Steele C.R., Lewis J.M., Glusac E., Filler R., Hobby P., Sutton B., Tigelaar R.E., Hayday A.C. (2001). Regulation of cutaneous malignancy by gammadelta T cells. Science.

[126] Alvarez A.E., Marson F.A., Bertuzzo C.S., Arns C.W., Ribeiro J.D. (2013). Epidemiological and genetic characteristics associated with the severity of acute viral bronchiolitis by respiratory syncytial virus. J. Pediatr. (Rio J).

[127] Janssen R., Bont L., Siezen C.L., Hodemaekers H.M., Ermers M.J., Doornbos G., van ’t Slot R., Wijmenga C., Goeman J.J., Kimpen J.L. (2007). Genetic susceptibility to respiratory syncytial virus bronchiolitis is predominantly associated with innate immune genes. J. Infect. Dis..

[128] Glass E.J., Baxter R., Leach R.J., Jann O.C. (2012). Genes controlling vaccine responses and disease resistance to respiratory viral pathogens in cattle. Vet. Immunol. Immunopathol..

[129] O’Neill R.G., Woolliams J.A., Glass E.J., Williams J.L., Fitzpatrick J.L. (2006). Quantitative evaluation of genetic and environmental parameters determining antibody response induced by vaccination against bovine respiratory syncytial virus. Vaccine.

[130] Kim H.W., Canchola J.G., Brandt C.D., Pyles G., Chanock R.M., Jensen K., Parrott R.H. (1969). Respiratory syncytial virus disease in infants despite prior administration of antigenic inactivated vaccine. Am. J. Epidemiol..

[131] Chin J., Magoffin R.L., Shearer L.A., Schieble J.H., Lennette E.H. (1969). Field evaluation of a respiratory syncytial virus vaccine and a trivalent parainfluenza virus vaccine in a pediatric population. Am. J. Epidemiol..

[132] Kapikian A.Z., Mitchell R.H., Chanock R.M., Shvedoff R.A., Stewart C.E. (1969). An epidemiologic study of altered clinical reactivity to respiratory syncytial (RS) virus infection in children previously vaccinated with an inactivated RS virus vaccine. Am. J. Epidemiol..

[133] Kimman T.G., Sol J., Westenbrink F., Straver P.J. (1989). A severe outbreak of respiratory tract disease associated with bovine respiratory syncytial virus probably enhanced by vaccination with modified live vaccine. Vet. Q..

[134] Schreiber P., Matheise J.P., Dessy F., Heimann M., Letesson J.J., Coppe P., Collard A. (2000). High mortality rate associated with bovine respiratory syncytial virus (BRSV) infection in Belgian white blue calves previously vaccinated with an inactivated BRSV vaccine. J. Vet. Med. B Infect. Dis. Vet. Public Health.

[135] West K., Petrie L., Haines D.M., Konoby C., Clark E.G., Martin K., Ellis J.A. (1999). The effect of formalin-inactivated vaccine on respiratory disease associated with bovine respiratory syncytial virus infection in calves. Vaccine.

[136] Mohanty S.B., Rockemann D.D., Davidson J.P., Sharabrin O.I., Forst S.M. (1981). Effect of vaccinal serum antibodies on bovine respiratory syncytial viral infection in calves. Am. J. Vet. Res..

[137] Mazur N.I., Higgins D., Nunes M.C., Melero J.A., Langedijk A.C., Horsley N., Buchholz U.J., Openshaw P.J., McLellan J.S., Englund J.A. (2018). The respiratory syncytial virus vaccine landscape: Lessons from the graveyard and promising candidates. Lancet Infect. Dis..

[138] Blanco J.C.G., Boukhvalova M.S., Morrison T.G., Vogel S.N. (2018). A multifaceted approach to RSV vaccination. Hum. Vaccin. Immunother..

[139] Villafana T., Falloon J., Griffin M.P., Zhu Q., Esser M.T. (2017). Passive and active immunization against respiratory syncytial virus for the young and old. Expert Rev. Vaccines.

[140] Hurwitz J.L. (2011). Respiratory syncytial virus vaccine development. Expert Rev. Vaccines.

[141] Yamazaki H., Tsutsumi H., Matsuda K., Nagai K., Ogra P.L., Chiba S. (1994). Effect of maternal antibody on IgA antibody response in nasopharyngeal secretion in infants and children during primary respiratory syncytial virus infection. J. Gen. Virol..

[142] Stensballe L.G., Ravn H., Kristensen K., Agerskov K., Meakins T., Aaby P., Simoes E.A. (2009). Respiratory syncytial virus neutralizing antibodies in cord blood, respiratory syncytial virus hospitalization, and recurrent wheeze. J. Allergy Clin. Immunol..

[143] Jans J., Wicht O., Widjaja I., Ahout I.M., de Groot R., Guichelaar T., Luytjes W., de Jonge M.I., de Haan C.A., Ferwerda G. (2017). Characteristics of RSV-Specific Maternal Antibodies in Plasma of Hospitalized, Acute RSV Patients under Three Months of Age. PLoS ONE.

[144] Freitas G.R., Silva D.A., Yokosawa J., Paula N.T., Costa L.F., Carneiro B.M., Ribeiro L.Z., Oliveira T.F., Mineo J.R., Queiroz D.A. (2011). Antibody response and avidity of respiratory syncytial virus-specific total IgG, IgG1, and IgG3 in young children. J. Med. Virol..

[145] Kimman T.G., Zimmer G.M., Westenbrink F., Mars J., van Leeuwen E. (1988). Epidemiological study of bovine respiratory syncytial virus infections in calves: Influence of maternal antibodies on the outcome of disease. Vet. Rec..

[146] Taylor G., Thom M., Capone S., Pierantoni A., Guzman E., Herbert R., Scarselli E., Napolitano F., Giuliani A., Folgori A. (2015). Efficacy of a virus-vectored vaccine against human and bovine respiratory syncytial virus infections. Sci. Transl. Med..

[147] Green C.A., Scarselli E., Sande C.J., Thompson A.J., de Lara C.M., Taylor K.S., Haworth K., Del Sorbo M., Angus B., Siani L. (2015). Chimpanzee adenovirus- and MVA-vectored respiratory syncytial virus vaccine is safe and immunogenic in adults. Sci. Transl. Med..

[148] Malkin E., Yogev R., Abughali N., Sliman J., Wang C.K., Zuo F., Yang C.F., Eickhoff M., Esser M.T., Tang R.S. (2013). Safety and immunogenicity of a live attenuated RSV vaccine in healthy RSV-seronegative children 5 to 24 months of age. PLoS ONE.

[149] Karron R.A., Wright P.F., Belshe R.B., Thumar B., Casey R., Newman F., Polack F.P., Randolph V.B., Deatly A., Hackell J. (2005). Identification of a recombinant live attenuated respiratory syncytial virus vaccine candidate that is highly attenuated in infants. J. Infect. Dis..

[150] Buchholz U.J., Cunningham C.K., Muresan P., Gnanashanmugam D., Sato P., Siberry G.K., Rexroad V., Valentine M., Perlowski C., Schappell E. (2018). Live Respiratory Syncytial Virus (RSV) Vaccine Candidate Containing Stabilized Temperature-Sensitivity Mutations Is Highly Attenuated in RSV-Seronegative Infants and Children. J. Infect. Dis..

[151] Sastry M., Zhang B., Chen M., Joyce M.G., Kong W.P., Chuang G.Y., Ko K., Kumar A., Silacci C., Thom M. (2017). Adjuvants and the vaccine response to the DS-Cav1-stabilized fusion glycoprotein of respiratory syncytial virus. PLoS ONE.

[152] McLellan J.S., Chen M., Joyce M.G., Sastry M., Stewart-Jones G.B., Yang Y., Zhang B., Chen L., Srivatsan S., Zheng A. (2013). Structure-based design of a fusion glycoprotein vaccine for respiratory syncytial virus. Science.

[153] Beran J., Lickliter J.D., Schwarz T.F., Johnson C., Chu L., Domachowske J.B., Van Damme P., Withanage K., Fissette L.A., David M.P. (2018). Safety and Immunogenicity of 3 Formulations of an Investigational Respiratory Syncytial Virus Vaccine in Nonpregnant Women: Results From 2 Phase 2 Trials. J. Infect. Dis..

[154] Kavanagh O.V., Adair B.M., Welsh M., Earley B. (2013). Immunogenetic responses in calves to intranasal delivery of bovine respiratory syncytial virus (BRSV) epitopes encapsulated in poly (DL-lactide-co-glycolide) microparticles. Res. Vet. Sci..

[155] Riffault S., Meyer G., Deplanche M., Dubuquoy C., Durand G., Soulestin M., Castagné N., Bernard J., Bernardet P., Dubosclard V. (2010). A new subunit vaccine based on nucleoprotein nanoparticles confers partial clinical and virological protection in calves against bovine respiratory syncytial virus. Vaccine.

[156] August A., Glenn G.M., Kpamegan E., Hickman S.P., Jani D., Lu H., Thomas D.N., Wen J., Piedra P.A., Fries L.F. (2017). A Phase 2 randomized, observer-blind, placebo-controlled, dose-ranging trial of aluminum-adjuvanted respiratory syncytial virus F particle vaccine formulations in healthy women of childbearing age. Vaccine.

[157] Fries L., Shinde V., Stoddard J.J., Thomas D.N., Kpamegan E., Lu H., Smith G., Hickman S.P., Piedra P., Glenn G.M. (2017). Immunogenicity and safety of a respiratory syncytial virus fusion protein (RSV F) nanoparticle vaccine in older adults. Immun. Ageing.

[158] Glenn G.M., Fries L.F., Thomas D.N., Smith G., Kpamegan E., Lu H., Flyer D., Jani D., Hickman S.P., Piedra P.A. (2016). A Randomized, Blinded, Controlled, Dose-Ranging Study of a Respiratory Syncytial Virus Recombinant Fusion (F) Nanoparticle Vaccine in Healthy Women of Childbearing Age. J. Infect. Dis..

[159] Blodorn K., Hagglund S., Fix J., Dubuquoy C., Makabi-Panzu B., Thom M., Karlsson P., Roque J.L., Karlstam E., Pringle J. (2014). Vaccine safety and efficacy evaluation of a recombinant bovine respiratory syncytial virus (BRSV) with deletion of the SH gene and subunit vaccines based on recombinant human RSV proteins: N-nanorings, P and M2-1, in calves with maternal antibodies. PLoS ONE.

[160] McLellan J.S., Chen M., Leung S., Graepel K.W., Du X., Yang Y., Zhou T., Baxa U., Yasuda E., Beaumont T. (2013). Structure of RSV fusion glycoprotein trimer bound to a prefusion-specific neutralizing antibody. Science.

[161] Graham B.S. (2017). Vaccine development for respiratory syncytial virus. Curr. Opin. Virol..

[162] Jorquera P.A., Tripp R.A. (2016). Synthetic Biodegradable Microparticle and Nanoparticle Vaccines against the Respiratory Syncytial Virus. Vaccines.

[163] Narasimhan B., Goodman J.T., Vela Ramirez J.E. (2016). Rational Design of Targeted Next-Generation Carriers for Drug and Vaccine Delivery. Annu. Rev. Biomed. Eng..

[164] Renukaradhya G.J., Narasimhan B., Mallapragada S.K. (2015). Respiratory nanoparticle-based vaccines and challenges associated with animal models and translation. J. Control. Release.

[165] Tran T.L., Castagné N., Bhella D., Varela P.F., Bernard J., Chilmonczyk S., Berkenkamp S., Benhamo V., Grznarova K., Grosclaude J. (2007). The nine C-terminal amino acids of the respiratory syncytial virus protein P are necessary and sufficient for binding to ribonucleoprotein complexes in which six ribonucleotides are contacted per N protein protomer. J. Gen. Virol..

[166] Hervé P.L., Deloizy C., Descamps D., Rameix-Welti M.A., Fix J., McLellan J.S., Eléouët J.F., Riffault S. (2017). RSV N-nanorings fused to palivizumab-targeted neutralizing epitope as a nanoparticle RSV vaccine. Nanomedicine.

[167] Roux X., Dubuquoy C., Durand G., Tran-Tolla T.L., Castagné N., Bernard J., Petit-Camurdan A., Eléouët J.F., Riffault S. (2008). Sub-nucleocapsid nanoparticles: A nasal vaccine against respiratory syncytial virus. PLoS ONE.

[168] Smith G., Raghunandan R., Wu Y., Liu Y., Massare M., Nathan M., Zhou B., Lu H., Boddapati S., Li J. (2012). Respiratory syncytial virus fusion glycoprotein expressed in insect cells form protein nanoparticles that induce protective immunity in cotton rats. PLoS ONE.

[169] Mills J., Van Kirk J.E., Wright P.F., Chanock R.M. (1971). Experimental respiratory syncytial virus infection of adults. Possible mechanisms of resistance to infection and illness. J. Immunol..

[170] Tsutsumi H., Matsuda K., Yamazaki H., Ogra P.L., Chiba S. (1995). Different kinetics of antibody responses between IgA and IgG classes in nasopharyngeal secretion in infants and children during primary respiratory syncytial virus infection. Acta. Paediatr. Jpn..

[171] Ellis J.A., Gow S.P., Goji N. (2010). Response to experimentally induced infection with bovine respiratory syncytial virus following intranasal vaccination of seropositive and seronegative calves. J. Am. Vet. Med. Assoc..

[172] Ellis J.A., Gow S.P., Mahan S., Leyh R. (2013). Duration of immunity to experimental infection with bovine respiratory syncytial virus following intranasal vaccination of young passively immune calves. J. Am. Vet. Med. Assoc..

[173] Tirabassi R.S., Ace C.I., Levchenko T., Torchilin V.P., Selin L.K., Nie S., Guberski D.L., Yang K. (2011). A mucosal vaccination approach for herpes simplex virus type 2. Vaccine.

[174] Yang K., Whalen B.J., Tirabassi R.S., Selin L.K., Levchenko T.S., Torchilin V.P., Kislauskis E.H., Guberski D.L. (2008). A DNA vaccine prime followed by a liposome-encapsulated protein boost confers enhanced mucosal immune responses and protection. J. Immunol..

[175] Dean G.S., Clifford D., Whelan A.O., Tchilian E.Z., Beverley P.C., Salguero F.J., Xing Z., Vordermeier H.M., Villarreal-Ramos B. (2015). Protection Induced by Simultaneous Subcutaneous and Endobronchial Vaccination with BCG/BCG and BCG/Adenovirus Expressing Antigen 85A against Mycobacterium bovis in Cattle. PLoS ONE.

[176] Lee C.K., Soike K., Giannasca P., Hill J., Weltzin R., Kleanthous H., Blanchard J., Monath T.P. (1999). Immunization of rhesus monkeys with a mucosal prime, parenteral boost strategy protects against infection with Helicobacter pylori. Vaccine.

[177] Tchilian E.Z., Ronan E.O., de Lara C., Lee L.N., Franken K.L., Vordermeier M.H., Ottenhoff T.H., Beverley P.C. (2011). Simultaneous immunization against tuberculosis. PLoS ONE.

[178] Jordan R., Shao M., Mackman R.L., Perron M., Cihlar T., Lewis S.A., Eisenberg E.J., Carey A., Strickley R.G., Chien J.W. (2015). Antiviral Efficacy of a Respiratory Syncytial Virus (RSV) Fusion Inhibitor in a Bovine Model of RSV Infection. Antimicrob. Agents Chemother..

[179] Walsh P., Behrens N., Carvallo Chaigneau F.R., McEligot H., Agrawal K., Newman J.W., Anderson M., Gershwin L.J. (2016). A Randomized Placebo Controlled Trial of Ibuprofen for Respiratory Syncytial Virus Infection in a Bovine Model. PLoS ONE.

[180] Cortjens B., de Jong R., Bonsing J.G., van Woensel J.B.M., Antonis A.F.G., Bem R.A. (2018). Local dornase alfa treatment reduces NETs-induced airway obstruction during severe RSV infection. Thorax.

